# Bacteriophage WO Can Mediate Horizontal Gene Transfer in Endosymbiotic *Wolbachia* Genomes

**DOI:** 10.3389/fmicb.2016.01867

**Published:** 2016-11-29

**Authors:** Guan H. Wang, Bao F. Sun, Tuan L. Xiong, Yan K. Wang, Kristen E. Murfin, Jin H. Xiao, Da W. Huang

**Affiliations:** ^1^Key Laboratory of Zoological Systematics and Evolution, Institute of Zoology, Chinese Academy of SciencesBeijing, China; ^2^University of Chinese Academy of SciencesBeijing, China; ^3^Disease Genomics and Individualized Medicine Laboratory, Beijing Institute of Genomics, Chinese Academy of SciencesBeijing, China; ^4^College of Life Sciences, Hebei UniversityBaoding, China; ^5^Section of Infectious Diseases, Yale University School of MedicineNew Haven, CT, USA

**Keywords:** horizontal gene transfer, bacteriophage WO, *Wolbachia*, obligate intracellular bacteria, transduction

## Abstract

Phage-mediated horizontal gene transfer (HGT) is common in free-living bacteria, and many transferred genes can play a significant role in their new bacterial hosts. However, there are few reports concerning phage-mediated HGT in endosymbionts (obligate intracellular bacteria within animal or plant hosts), such as *Wolbachia*. The *Wolbachia*-infecting temperate phage WO can actively shift among *Wolbachia* genomes and has the potential to mediate HGT between *Wolbachia* strains. In the present study, we extend previous findings by validating that the phage WO can mediate transfer of non-phage genes. To do so, we utilized bioinformatic, phylogenetic, and molecular analyses based on all sequenced *Wolbachia* and phage WO genomes. Our results show that the phage WO can mediate HGT between *Wolbachia* strains, regardless of whether the transferred genes originate from *Wolbachia* or other unrelated bacteria.

## Introduction

Horizontal gene transfer (HGT), or lateral gene transfer, is the exchange of genetic elements across species. Abundant evidence of HGT has been detected over the last few decades, particularly in prokaryotic organisms (Ortiz et al., 2015). The genes acquired by HGT can provide new activities to a bacterial host (Waldor and Mekalanos, 1996; Brüssow et al., 2004; Rodriguez-Valera et al., 2009; Modi et al., 2013). Additionally, these genes can play a significant role in the ecological and evolutionary adaptation to a new host (Ochman et al., 2000). Bacteriophages, plasmids, and transposons are the typical genetic vehicles that mediate HGT (Brüssow et al., 2004). The global rate of phage-mediated HGT events is estimated to be as much as 2 × 10^16^ per second (Bushman, 2002). Recently, molecular evidence for HGT in the genomes of several obligate intracellular bacteria has been reported (Gavotte et al., 2004; Ishmael et al., 2009; Chafee et al., 2010). However, the role of phage in such transfers has not been thoroughly investigated and is considered likely to be rare due to the constraints of an intracellular lifestyle (Fineran et al., 2009).

The obligate intracellular bacterium *Wolbachia*, a cytoplasmically inherited Rickettsiales, has recently attracted increasing attention. As one of the most widespread endosymbionts in nature (Hilgenboecker et al., 2008; Zug and Hammerstein, 2012), *Wolbachia* can manipulate arthropod hosts' reproductive systems to facilitate their own spread (Werren et al., 2008). Accordingly, there is worldwide interest in using *Wolbachia*-infected mosquitoes to reduce mosquito populations for the elimination of mosquito-borne pathogens, such as dengue virus (Zabalou et al., 2004; Turley et al., 2009; Walker et al., 2011). *Wolbachia* also has a mutualistic relationship with filarial nematodes and is a potential drug target for filarial diseases (Nutman, 2001; Taylor et al., 2001). However, studies on the applications of *Wolbachia* have been seriously hampered due to the lack of *in vitro* culture methods and genetic transformation tools for testing *Wolbachia* gene function (Fujii et al., 2004). The phage WO, which can infect *Wolbachia*, has the potential to mediate gene transfer and thus offers hope for *Wolbachia* transformation and genetic engineering (Fujii et al., 2004; Metcalf and Bordenstein, 2012).

In this study, we investigated the hypothesis that phage WO might mediate HGT in *Wolbachia.* Several considerations support this hypothesis. First, as a temperate phage that can shift between the lysogenic and lytic forms, phage WO is a dynamic element in the *Wolbachia* genome (Masui et al., 2001). Second, phage WO is widespread among *Wolbachia* genomes (present in about 89%; Bordenstein and Wernegreen, 2004). Nearly all sequenced *Wolbachia* genomes, if infected with phage WO, have at least one intact WO prophage (Kent et al., 2011a), which has the potential to produce phage particles. Third, the transfer of the phage minor capsid gene (Masui et al., 2001; Bordenstein and Wernegreen, 2004; Gavotte et al., 2007; Chafee et al., 2010) and the complete bacteriophage (Kent et al., 2011b) has been observed between different *Wolbachia* strains. All of the above indicate that other genetic material associated with phage WO may also be transferred when phage WO transfers between hosts.

We used bioinformatic, molecular, and phylogenetic analyses of all the published *Wolbachia* and phage WO genomes to investigate the occurrence of phage WO mediated HGT in *Wolbachia*. We first detected the “alien” genes associated with phage WO through blastp and blastn searches, phylogenetic approaches (genes with restricted distributions) or parametric approaches (genes showing distinct nucleotide composition bias or molecular evolution pattern compared to bacterial host genes). These “alien” genes are shown to be packaged in phage WO by a combination of experimental evidence, molecular experiments of reverse PCR or real-time qPCR, and from literature searching. However, these phylogenetic and parametric approaches do not suggest that these “alien” genes are of virus origin (Azad and Lawrence, 2012). In addition, thorough comparable genomic analyses are used to investigate phage WO horizontal transfer vestiges and their association with mediating transfer of “alien” genes.

## Materials and methods

### Data mining

The complete prophage WOcauB3 (B3gp1–B3gp46), prophage WOcauB2 (B2gp1–B2gp47), the flanking-region genes from the prophage WOVitA1 (VA1gp52–VA1gp63), and two flanking-region genes from the prophage WORiB1 (WRi_005400–WRi_005900) were used as queries in a blastp search of the NCBI non-redundant protein database and a blastn search of the NCBI nucleotide collection (nr/nt) and whole-genome shotgun contigs (wgs) databases. The output *E*-value (<10^−5^) of the searches were used as criteria for data parsing. Sequences were aligned with ClustalW in BioEdit (Hall, 1999), and the Gblocks program (ver. 0.91b) (Castresana, 2000) was used to remove poorly aligned positions.

### Phylogenetic analysis

For phylogenetic analyses, ProtTest 3 (for amino acid sequences; Darriba et al., 2011) and jModelTest 2 (for nucleotide sequences; Darriba et al., 2012) were used to determine the best evolution model based on the corrected Akaike information criterion (AICc). PhyML 3.0 (Guindon et al., 2010) and Mrbayes 3.2 (Ronquist et al., 2012) were used to build phylogenetic trees with ML and BI methods respectively. The best models chosen by ProtTest 3, LG + I + G was used to generate the ML and BI tree for B3gp45. The best model chosen by jModelTest 2, GTR+G, was used to generate the ML tree for the *Wolbachia* MLST phylogeny.

### Sequence analysis

To visualize the general compositional features of the putative horizontally transferred genes using GC-content, a cumulative GC profile was assembled (Gao and Zhang, 2006). The cumulative GC profile can identify genomic islands or HGTs through comparison of nucleotide compositional features (Gao and Zhang, 2006). The halting parameter was set to 7, and the minimum length to segment was set to 100.

### Selection analysis

MEGA6 was used to estimate the mean synonymous divergence for each group of sequences representing potential recent horizontal transfer of WO phages, other WO phages that seem not to results from recent horizontal transfer, and their corresponding *Wolbachia* hosts (Tamura et al., 2013). For each group of sequences, the Nei-Gojobori method was used to calculate the synonymous rate, and variance was computed using 1000 bootstrap replicates (Nei and Gojobori, 1986).

### Sample collection

*Musca domestica* and *Nasonia vitripennis* were used in these experiments. The *N. vitripennis* populations were the Hangzhou strain (from the Gongyin Ye lab, ZheJiang University) (Zhang et al., 2005) infected with *Wolbachia* supergroup A (Liu et al., 2014). The housefly larvae were fed bran for 5–6 days until pupation. All wasps were reared on fresh house fly pupae at 25 ± 2°C under a 14 h light cycle in an atmosphere of 50–60% relative humidity, supplemented with a piece of cotton in a soft capsule shell of 10% honey water. The adult houseflies were kept at 25 ± 2°C, but supplied with a sugar/milk powder mixture (25/75%) and water instead. Adults of *N. vitripennis* were initially immersed in 95% ethanol at −20°C prior to DNA extraction.

### DNA extraction, PCR amplification and cloning

Total *N. vitripennis* genomic DNA was extracted from a single wasp using the DNeasy Tissue Kit (Qiagen, Hilden, Germany) following the manufacturer's recommendations and resuspended in 20 μl double-distilled sterile water. DNA purity and concentration were determined with a NanoDrop 2000 Spectrophotometer (Thermo, Madison, WI, USA), and samples of poor quality were discarded. The identity of the DNA templates was confirmed by *wsp* 81f and 691r primers to amplify the *Wolbachia* surface protein gene (Zhou et al., 1998). The PCR reactions were performed using TransTaq DNA Polymerase HiFi Fidelity (TransGen Biotech, Beijing, China) with the recommended conditions and reagents. The resulting amplicons were electrophoresed on a 1% TBE agarose gel and photographed under UV illumination. The amplified PCR products were sequenced directly with an ABI3730 capillary autosequencer (Biosune, Beijing, China) after purification with the EasyPure PCR Purification Kit (TransGen Biotech, Beijing, China). If the products could not be sequenced directly, we cloned them into the pEASY-T5 vector (TransGen Biotech, Beijing, China), and a minimum of three positive clones were sequenced due to transformation-induced mutation. Sequence editing was performed with BioEdit (Hall, 1999).

### Real-time qPCR

Real-time qPCR was performed with a Stratagene Mx3000p qPCR System (Stratagene, La Jolla, CA, USA) (the primers are listed in Table S3). We used real-time qPCR to quantify the DNA copies of a putative transcriptional regulator gene (VA1gp53) and an Hsp20-family heat shock protein gene (VA1gp62) from the flanking region of WOVitA1; an *ank* gene (VA1gp3) from phage WOVitA1; and a heat-shock protein 60 gene (*groEL*) (Bordenstein et al., 2006) and cell division gene (*ftsZ*) from *w*VitA vs. prepared standard solutions. The amplified PCR products were sequenced directly to confirm the gene identity. A standard 10-fold dilution series from 10^7^ to 10^3^ copies were prepared and used to calculate the copy numbers of the genes. The genes's amplification efficiency in our experiments are 96.6–104.4%. Also, each melting curve showed that the primers amplify a single product.

### Statistical analysis

The average copy number of the integrated phage was compared with the expected number and the difference was analyzed statistically with a two-tailed *t*-test (SAS Institute, Cary, NC, USA). With a single lysogenic copy of WOVitA1, the expected WOVitA1 number should always equal (no lytic activity) or exceed (with lytic activity producing multiple phage virions) the *w*VitA copy number. We normalized the small plate effects in real-time qPCR experiments as described previously (Wang et al., 2014). The compared percent nucleotide identity was analyzed by an Mann–Whitney U two-tailed test using Origin8.0.

### Nucleotide sequence accession number

*De novo* nucleotide sequences were deposited in GenBank under accession numbers KP966832–KP966840.

## Results

**Figure 1:**
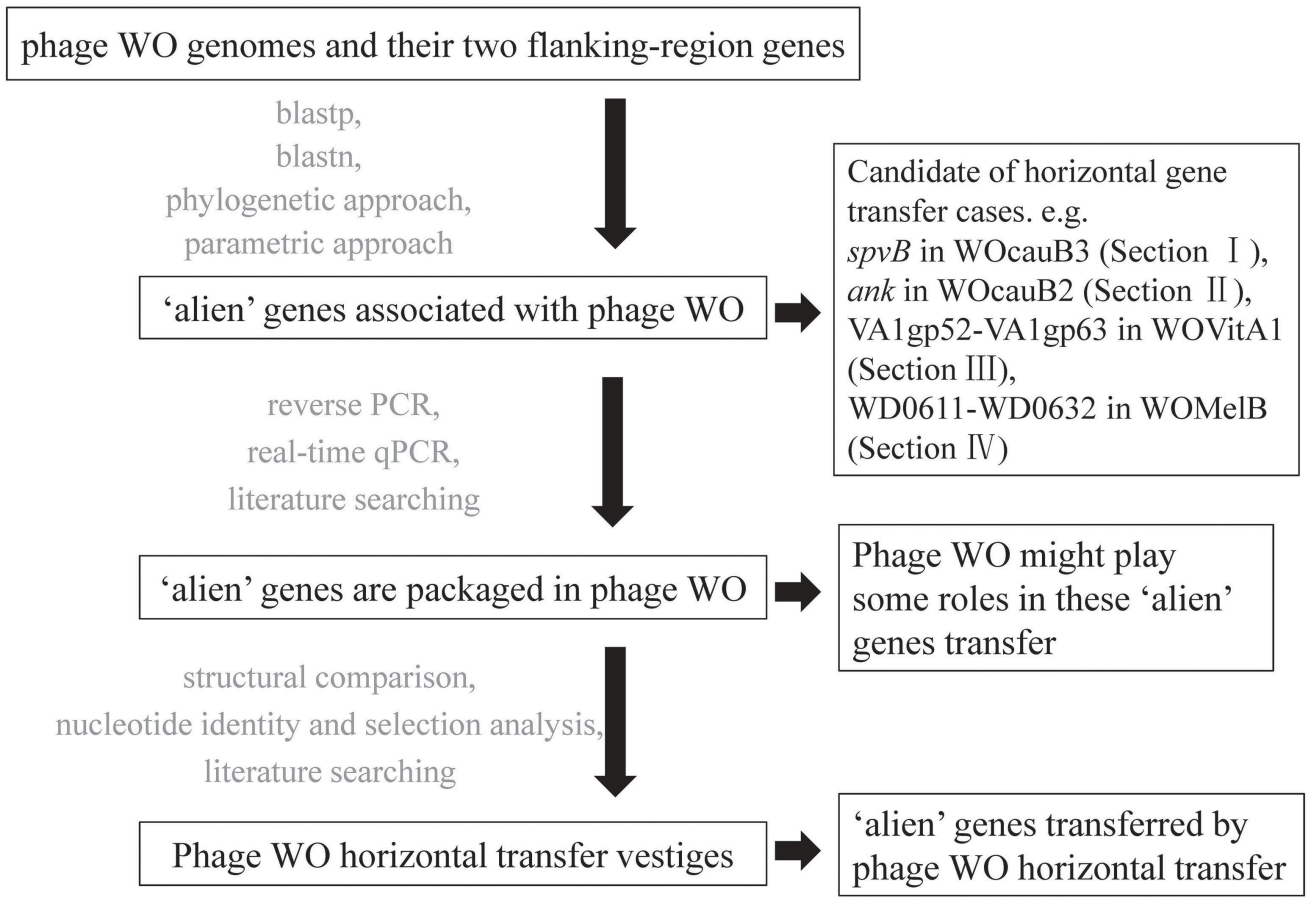
**Flow chart of the screening methods and the results of each step used to detect phage WO mediating horizontally transferred genes**.

Several previous studies have shown that the phage WO might mediate HGT. The genome of the *Wolbachia* endosymbiont (*w*CauB) of the flour moth, *Ephestia kuehniella*, contains two related prophages, WOcauB2 and WOcauB3 (Table 1), which share high nucleotide sequence identity and conserved gene arrangements (Tanaka et al., 2009). However, there are differences in the 3′ ends of both phages: two ankyrin-domain-containing (*ank*) genes (B2gp46 and B2gp47) are present in WOcauB2 but absent in WOcauB3. Additionally, WOcauB3 possesses a *Salmonella* virulence plasmid protein B gene (B3gp45, *spvB* gene) and a hypothetical protein-encoding gene (B3gp46) that WOcauB2 lacks. These differences indicate that though quite similar, WOcauB2 and WOcauB3 are mobile elements that have experienced dynamic evolutionary trajectories. The genes only present in WOcauB2 (e.g., *ank*) or WOcauB3 (e.g., *spvB*) are suggested to have been transduced by phage WO (Tanaka et al., 2009). Occasionally, the transfer of a complete phage can occur between different *Wolbachia* strains. For example, the WO phage WOVitA1 can transfer between *Wolbachia w*VitA and *w*VitB strains hosted in *N. vitripennis*, and interestingly, the transfer seems to involve not only the phage region (including genes of VA1gp1–VA1gp51) but also the flanking bacterial region (Kent et al., 2011b). In this work, we used a series of stringent filters to identify phage WO mediating HGT events (Figure 1).

**Table 1:** **The sequenced prophage and ***Wolbachia*** genomes**.

**Prophage**	***Wolbachia***	**Phenotype**	**Host**	**Common name**	**Supergroup**	**Status^a^**	**Region**	**References**
WOcauB1	*w*CauB	CI	*Ephestia kuehniella*	moth	B	Unfinished	gp1~gp24	Fujii et al., 2004
WOcauB2							B2gp1~B2gp47	Tanaka et al., 2009
WOcauB3							B3gp1~B3gp46	
WONo1	*w*No	CI	*D. simulans*	fruit fly	B	Complete	*w*No_01060~*w*No_01380	Ellegaard et al., 2013
WONo2							*w*No_07250~*w*No_07370	
WONo3							*w*No_09030~*w*No_09160	
WONo4							*w*No_10080~*w*No_10280	
WORiA	*w*Ri	CI	*D. simulans*	fruit fly	A	Complete	*W*Ri_012450~*W*Ri_012670	Klasson et al., 2009
WORiB1							WRi_005400~WRi_005720	
WORiB2							WRi_010060~WRi_010380	
WORiC							WRi_006880~WRi_007250	
WOVitA1	*w*VitA	CI	*Nasonia vitripennis*	jewel wasp	A	Unfinished	VA1gp1~VA1gp51	Kent et al., 2011b
WOVitA2							VA2gp1~VA2gp39	
WOVitA4							VA4gp1~VA4gp28	
WOVitB	*w*VitB	CI	*N. vitripennis*	jewel wasp	B	Unfinished	HQ906665	Kent et al., 2011b
WOSol1	*w*Cs	Unknown	*Ceratosolen solmsi*	fig wasp	A	Unfinished	So0001~So0025	Wang et al., 2013
WOSol2							So0026~So0029	
WOMelA	*w*Mel	CI	*Drosophila melanogaster*	fruit fly	A	Complete	WD0259~WD0292	Wu et al., 2004
WOMelB1							WD0565~WD0610	
WOMelB2							WD0633~WD0644	
WOMelPop (partial)	*w*MelPop	CI	*D. melanogaster*	fruit fly	A	Unfinished	contig_00005_6 1056~49398	Woolfit et al., 2013
WOSuz1	*w*lb_suzi	Unknown	*D. suzukii strain* DS-VAL-F5	fruit fly	A	Unfinished	contig005 19344~41162	Siozios et al., unpublished
WOSuz2							contig014 35799~42456	
WOSuz3							contig024	
WOAuA	*w*Au	non CI	*D. simulans*	fruit fly	A	Complete	WPWAU0631~WPWAU0666	Sutton et al., 2014
WOAuB							WPWAU0282~WPWAU0318	
WOMol1	*w*PipMol	CI	*C. molestus*	mosquito	B	Unfinished	WPM_000998~WPM_001001	Pinto et al., 2013
WOMol2							WPM001007c~WPM_001048	
WOMol3							WPM_001076~WPM_001092	
WOMol4							WPM001101c~WPM_001163	
WOMol5							WPM_001164~WPM_001190	
WOHa1	*w*Ha	CI	*D. simulans*	fruit fly	A	Complete	wHa02360~wHa02660	Ellegaard et al., 2013
WOHa2							wHa03390~wHa03840	
WOPip1	*w*Pip Pel	CI	*Culex pipiens*	mosquito	B	Complete	WP0242~WP0272	Klasson et al., 2008
WOPip2							WP0297~WP0322	
WOPip3							WP0323~WP0342	
WOPip4							WP0411~WP0455	
WOPip5							WP1294~WP1340	
–	*w*Oo	mutualism	*Onchocerca ochengi*	nematode	C	Complete	–	Darby et al., 2012
–	*w*Ov	mutualism	*O. volvulus*	nematode	C	Complete	–	Desjardins et al., 2013
–	*w*Bm	mutualism	*Brugia malayi*	nematode	D	Complete	–	Foster et al., 2005
–	*w*Cle	mutualism	*Cimex lectularius*	bug	F	Complete	–	Nikoh et al., 2014
	*w*Ana	CI	*D. ananassae*	fruit fly	A	Unfinished		Salzberg et al., 2005
	*w*Sim	CI presumed	*D. simulans*	fruit fly	A	Unfinished		Salzberg et al., 2005
	*w*Moj	Unknown	*D. mojavensis*	fruit fly	A	Unfinished		Salzberg et al., 2005
	*w*Uni	Parthenogenesis	*Muscidifurax uniraptor*	wasp	A	Unfinished		Klasson et al., 2009
WORec A	*w*Rec	CI (male killing)	*D. recens* (*D. subquinaria*)	fruit fly	A	Unfinished	WREC0261 ~WREC0285	Metcalf et al., 2014
WORec B							WREC0559 ~WREC 0568	
	*w*Gmm	CI	*Glossina morsitans*	tsetse fly	A	Unfinished		Brelsfoard et al., 2014
	*w*Coc	Unknown	*Dactylopius coccus*	cochineal	A	Unfinished		Campana et al., 2015
	*w*Wil	Unknown	*D. willistoni*	fruit fly	A	Unfinished		Salzberg et al., 2005
	*w*Pip JHB	CI	*C. quinquefasciatus* JHB	mosquito	B	Unfinished		Salzberg et al., 2009
	*w*AlbB	CI	*Aedes albopictus*	mosquito	B	Unfinished		Mavingui et al., 2012
	*w*Di	Unknown	*Diaphorina citri*	bug	B	Unfinished		Saha et al., 2012
	*w*Bol1	Male killing	*Hypolimnas bolina*	butterfly	B	Unfinished		Duplouy et al., 2013
	*w*Wb	Unknown	*Wuchereria bancrofti*	nematode	D	Unfinished		Desjardins et al., 2013

a Wolbachia genome assembly information.

CI: Cytoplasmic incompatibility. –: None. Blank space: no statistics because of poor genome assembly.

### Phage WOcauB3 has transferred between *W*cauB and wNo (A *wolbachia* strain from supergroup B infecting D. *simulans*) and mediated gene transfer

**Figure 2:**
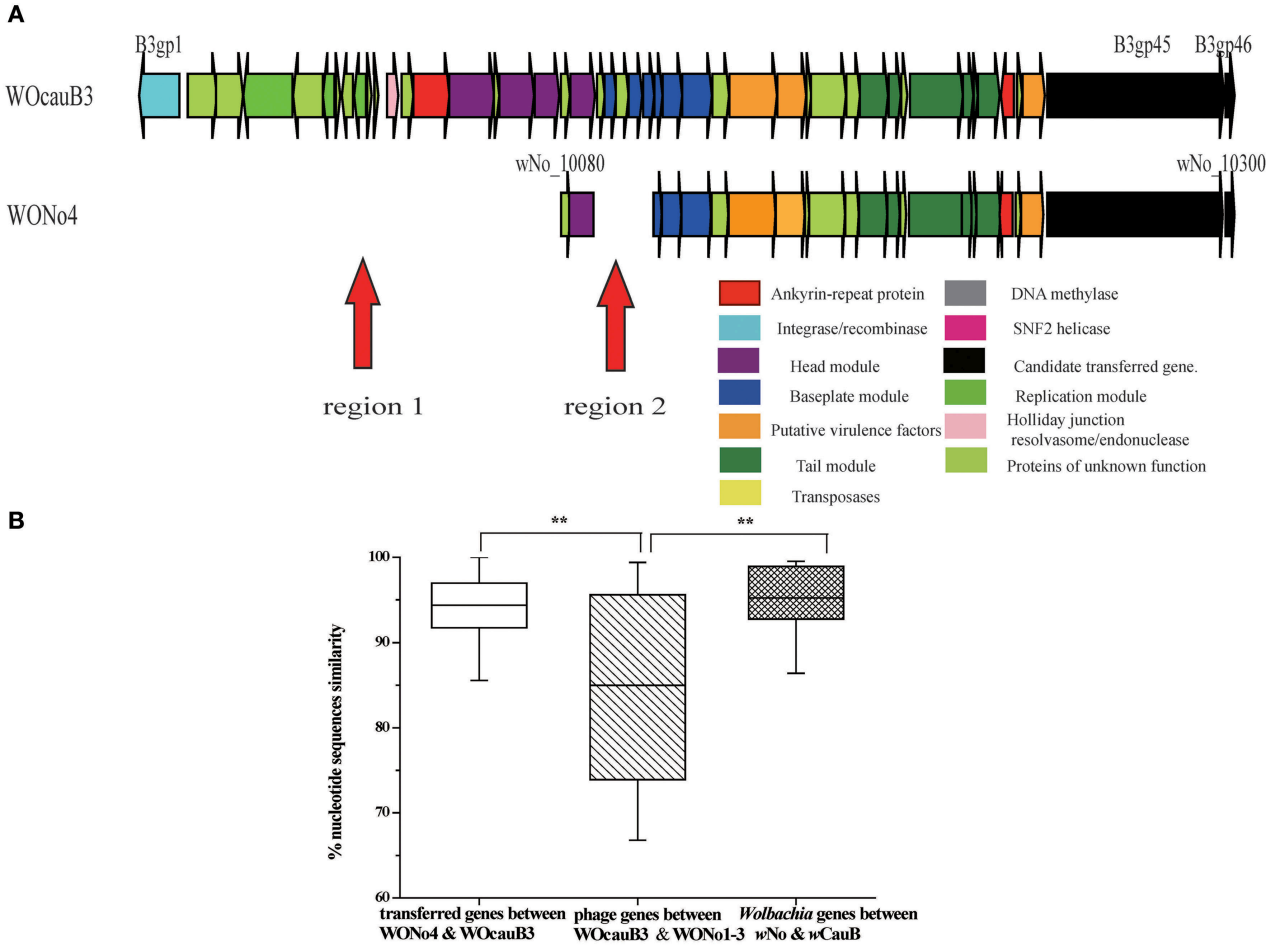
**Phage WO transferred between ***w***CauB and ***w***No. (A)** Structural comparison between the WOcauB3 and WONo4 prophage sequences. Red arrows indicate discrepant regions between the prophage genomes. Genes are presented by arrows while psudogenes and non-coding regions are boxes. Genes are colored based on functional type and homology. **(B)** Percent nucleotide identity between prophage genes encoded on WOcauB3, WONo4, and other phages. Percent nucleotide identity is compared between phage genes transferred from WOcauB3 (between WOcauB3 and WONo4), other phage genes (between WOcauB3 and WONo1, WONo2, WONo3 in *w*No that are likely not transferred from WOcauB3), *Wolbachia* genes (*w*No and *w*CauB previously sequenced protein-coding genes). Error bars represent one standard deviation. The double asterisk indicates a significant difference (*P* <0.01; Mann–Whitney U, two-tailed test).

Previous searches of public databases suggested the transfer of bacterial *spvB* gene (B3gp45) between an unrelated bacterial genetic lineage and *Wolbachia w*CauB by WO (Tanaka et al., 2009). In the present study, we expand this finding by conducting a homology search for the complete phage WOcauB3 genes in all of the 32 reported *Wolbachia* genomes (information on all *Wolbachia* genomes in this study is listed in Table 1). Of the 32 genomes tested, we detected that only two, *w*CauB and *w*No, have uniquely encoded *spvB* and nearby gene, B3gp46, which encodes a hypothetical protein that is packaged in phage WOcauB3 particles (Tanaka et al., 2009; Figure 2A). The *Wolbachia w*No is a strain from supergroup B infecting *D. simulans* (Ellegaard et al., 2013).

To further trace the transfer trajectory of *spvB* and B3gp46, we compared the divergence between prophages WOcauB3 and WONo1–4 and between their *Wolbachia* hosts, *w*CauB and *w*No. The *Wolbachia* strain *w*No harbors four WO phages, WONo1–4 (Table 1).

Several lines of evidence support the possibility that WOcauB3 was transferred between *w*CauB and *w*No and mediated the transfer of both genes.

#### Structural comparisons of WOcauB3 with WONo4 or with WONo1,3

Structurally, the genes in prophages WOcauB3 and WONo4 are syntenic, except in two regions: region 1 (including B3gp1–B3gp18) and region 2 (B3gp21–B3gp25). These regions are present in WOcauB3 and absent in WONo4 (Figure 2A). However, when WOcauB3 is compared with prophages WONo1 or WONo3 (WONo2 is not included in the analysis due to its short length), the gene orders are only partially conserved, indicating frequent inversion/translocation/recombination events (Figure S1A). This structural pattern indicates a recent transfer between WOcauB3 and WONo4 with the erosion of recombination, replication, head, and baseplate module as few genes exist in these modules in WONo4 while are present in WOcauB3.

#### Nucleotide identity and selection analyses between WOcauB3 and WONo4, WOcauB3, and WONo1–3, and their wolbachia hosts wCauB and wNo

Overall, prophage WOcauB3 genes are 94.37% identity to those of WONo4 at the nucleotide level (range 83.74–100.00%), which is significantly higher than the average 84.97% nucleotide identity between WOcauB3 and the other phages (WONo1–3) in the *w*No genome [range 66.78–99.39%; Mann–Whitney U (MWU), two-tailed, *P* < 0.01; Figure 2B]. In addition, the synonymous mutation rate between prophage WOcauB3 and WONo4 is 0.07 (range 0.00–0.48), significantly lower than the average 0.27 between WOcauB3 and phages WONo1–3 (range 0.00–0.61; MWU, two-tailed, *P* < 0.01, data not shown). This also demonstrates a smaller divergence between prophage WOcauB3 and WONo4 than between WOcauB3 and WONo1–3. Additionally, the sequenced *Wolbachia* protein-coding genes (Table S1) from *w*CauB and *w*No have a significantly higher nucleotide identity (95.74%, range 83.18–99.53%) than the phages WOcauB3 and WONo1–3 (MWU, two-tailed, *P* < 0.01; Figure 2B). It is noteworthy that the average nucleotide identity of WOcauB3 and WONo4 is not significantly different than the average nucleotide identity of *Wolbachia* genes from *w*CauB and *w*No (MWU, two-tailed, *P* >0.05; Figure 2B). Given the 3.5-fold higher sequence diversity between WOcauB3 and WONo1–3 when compared to *w*CauB and *w*No, it conservatively indicates that phage WOcauB3 may transfer directly from *w*CauB to *w*No, or indirectly through other unsequenced *Wolbachia* hosts to *w*No. Also, the synonymous mutation rate between WOcauB3 and WONo4 (0.07) is not significantly different from that between *Wolbachia* protein-coding genes of *w*CauB and *w*No (0.10, range 0.01–0.29; MWU, two-tailed, *P* > 0.05, data not shown). However, there is approximately a 3.0-fold higher synonymous mutation rate between WOcauB3 and WONo1–3, when compared with *w*CauB and *w*No protein-coding genes.

Furthermore, a *Wolbachia* phylogenetic tree constructed using the Multi-Locus Sequence Typing (MLST) method indicates that *w*No and *w*CauB are not closely related *Wolbachia* strains (Figure S2). If phages WOcauB3 and WONo4 were assumed vertically descended from a recent common ancestor, it would require at least three independent losses (based on Figure S2 phylogenetic tree) of this phage in *Wolbachia* strains of *w*VitB, *w*PipPel (infecting *Culex pipiens*; Klasson et al., 2008), and *w*PipMol (infecting *Culex molestus*; Pinto et al., 2013), which is less parsimonious than a single phage horizontal transfer event. All of the above analyses suggest that WOcauB3 was horizontally transferred. Previous reports have demonstrated that *w*No infects *D. simulans* (Ellegaard et al., 2013) and *w*CauB infects *E. kuehniella* (Tanaka et al., 2009). Therefore, it seems likely that these *Wolbachia* strains infect an intermediate host concurrently to facilitate exchange of the phage WOcauB3.

#### SpvB and nearby B3gp46 gene are transferred via the transmission of WOcauB3

**Figure 3:**
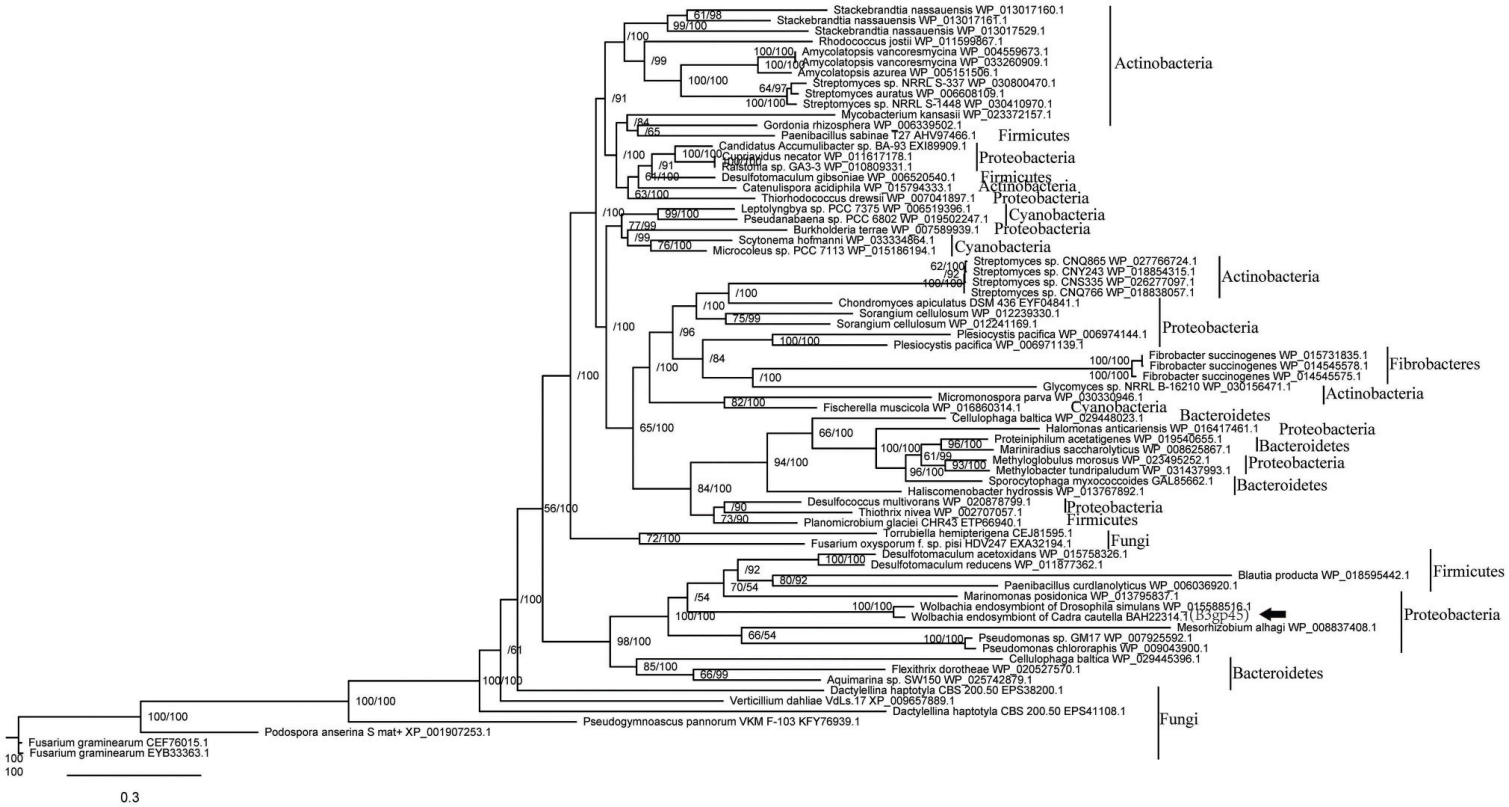
**Phylogeny based on the amino acid sequences of phage WOcauB3 ***spvB*** gene B3gp45 and its homologs**. Phylogeny based on alignment of 766 aa consisting of top *E*-value (<10^−5^) hits to blastp using WOcauB3 *spvB* gene as a query. There is an apparent transfer from unrelated genetic lineages of bacteria to *Wolbachia*, marked with arrowhead. Each tip is labeled with the species name and the sequence's Genbank accession number. Bootstrap values (maximum likelihood phylogeny) and posterior probability (bayesian phylogeny) higher than 50% are shown. The black box on the right represents the phyla.

Homologs of *spvB* (B3gp45) and nearby B3gp46 gene from WOcauB3 are present in only two of the 32 sequenced *Wolbachia* strains, *w*CauB and *w*No (Figure 2A). *spvB* phylogenetic tree inferred from Maximum Likelihood (ML) and Bayesian Inference (BI) methods (Figure 3) is shown. In public databases, there are no closely related orthologs of B3gp46 except in the *w*CauB and *w*No. These scattered distribution patterns indicate recent transmission of the *spvB* and B3gp46 genes. Furthermore, both genes are located at the 3′ ends of phages WOcauB3, WONo4 (Figure 2A) and are packaged into WOcauB3 (Tanaka et al., 2009), indicating that phage WO is the vehicle of their transmission.

### Phage WOcauB2 has transferred between wCauB and wRi and mediated the horizontal transfer of two *ank* genes

In addition to WOcauB3, there is also evidence to support that WOcauB2 (from the same *Wolbachia* strain, *w*CauB) has experienced a transmission event. The transmission likely mediated the horizontal transfer of two associated *ank* genes.

#### Structural comparison of WOcauB2 with WORiC or with WORiA and WORiB1

**Figure 4:**
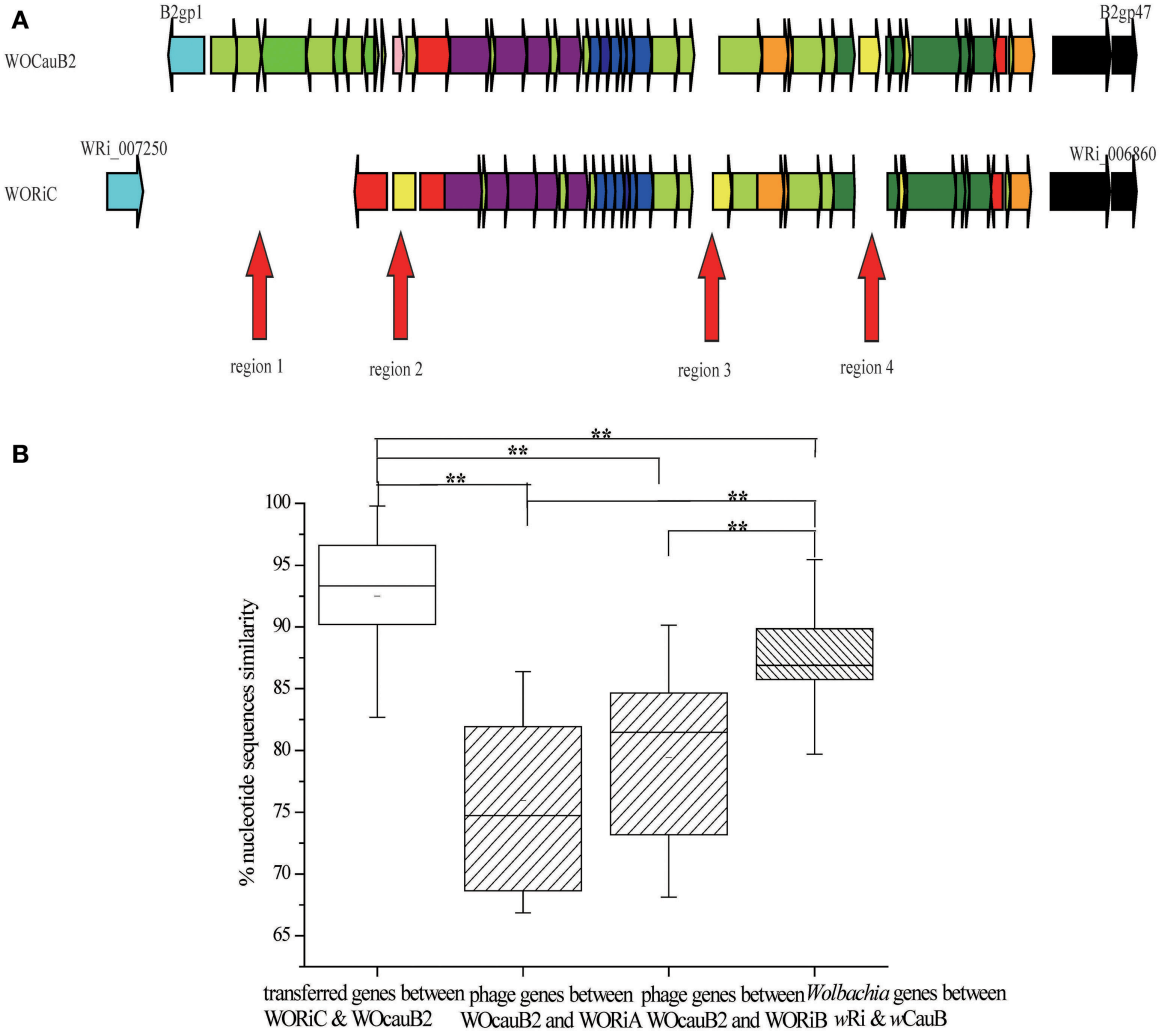
**Phage WO transferred between ***w***CauB and ***w***Ri. (A)** Structural comparison between the WOcauB2 and WORiC prophage sequences. Red arrows indicate discrepant regions between the prophage genomes. Genes are presented by arrows while psudogenes and non-coding regions are boxes. Colors of ORFs are as described in the legend of Figure 2. **(B)** Percent nucleotide identity between prophage genes encoded on WOcauB2, WORiC, and other phages. Percent nucleotide identity is compared between phage genes transferred from WOcauB2 (between WOcauB2 and WORiC), other phage genes (between WOcauB2 and WORiA and WORiB in wRi that are unlikely transferred from WOcauB2), and Wolbachia genes (previously sequenced protein-coding genes in *Wolbachia w*CauB and wRi). Error bars represent one standard deviation. The double asterisk indicates a significant difference (*P* <0.01; Mann–Whitney U, two-tailed test).

The prophages WOcauB2, from *Wolbachia w*CauB, and WORiC, from *Wolbachia w*Ri, are syntenically conserved with the exception of four heterogeneous regions, including a deletion of the B2gp2–B2gp12 region in WORiC (region 1), two insertions of transposase genes (WRi_007230 and WRi_007040; regions 2 and 3), and a deletion of a transposase gene (B2gp35) in WORiC (region 4; Figure 4A). However, gene order between WOcauB2 and WORiA or WORiB1, also from *Wolbachia w*Ri (Klasson et al., 2009), are only partially conserved (Figure S1B; WORiB1 and WORiB2 are identical, so only WORiB1 is used for analysis in this study; Ishmael et al., 2009; Tanaka et al., 2009; Wang et al., 2013). Taken together, these similarities indicate a phage transfer (WOcauB2 and WORiC) between *w*CauB and *w*Ri.

#### Nucleotide identity and selection analysis between WOcaub2 and WORiC, WOcauB2 and WORiA or WORiB1, and their wolbachia hosts wCauB and wRi

Comparison of nucleotide identity revealed that, on average, WORiC genes are 92.52% nucleotide identity to genes of the phage WOcauB2 (range 77.98–99.79%), which is significantly higher than the average nucleotide identity between WOcauB2 and the other phages in the *w*Ri genome: WORiA shares 75.94% nucleotide identity with WOcauB2 (range 66.87–86.39%), and WORiB1 shares 79.43% nucleotide identity with WOcauB2 (range 68.15–90.15%; MWU, two-tailed, *P* < 0.01; Figure 4B). The nucleotide identity between WORiC and WOcauB2 genes is also significantly higher than the average nucleotide identity between the sequenced *Wolbachia* protein-coding genes (Table S2) from *w*CauB and *w*Ri (86.78%; range 68.26–98.71%; MWU, two-tailed, *P* < 0.01; Figure 4B). Additionally, the nucleotide identity between *Wolbachia* strains (86.78%) is significantly higher than identity between WOcauB2 and WORiA (75.94%) or phage WORiB1 (79.43%; MWU, two-tailed, *P* < 0.01; Figure 4B). These results suggest that phage WOcauB2 may transfer directly from *w*CauB to *w*Ri, or indirectly through other unsequenced *Wolbachia* hosts to *w*Ri. Furthermore, the synonymous mutation rate between prophages WOcauB2 and WORiC is 0.13 (range 0.00–0.53), significantly lower than that between WOcauB2 and the phages WORiA (0.34; range 0.10–0.63) and WORiB1 (0.22; range 0.04–0.58) in the *w*Ri genome (MWU, two-tailed, *P* < 0.01; data not shown). The synonymous mutation rate between prophages WOcauB2 and WORiC is also lower than that between *w*CauB and *w*Ri (0.32; range 0.04–0.56; MWU, two-tailed, *P* < 0.01; data not shown).

Considering that *w*Ri belongs to the *Wolbachia* supergroup A and *w*CauB belongs to supergroup B (Figure S2), this distant phylogenetic relationship excludes the possibility that WOcauB2 and WORiC are descended from a recent common ancestor. This further supports the transfer of WOcauB2 between *w*CauB and *w*Ri. As previously reported, *w*Ri infects *D. simulans* (Klasson et al., 2009) and *w*CauB infects *E. kuehniella* (Tanaka et al., 2009), which suggests that the exchange of phage WOcauB2 may have been facilitated by the coninfection of an intermediate host.

#### Two ank genes are transferred via the transmission of WOcauB2

**Figure 5:**
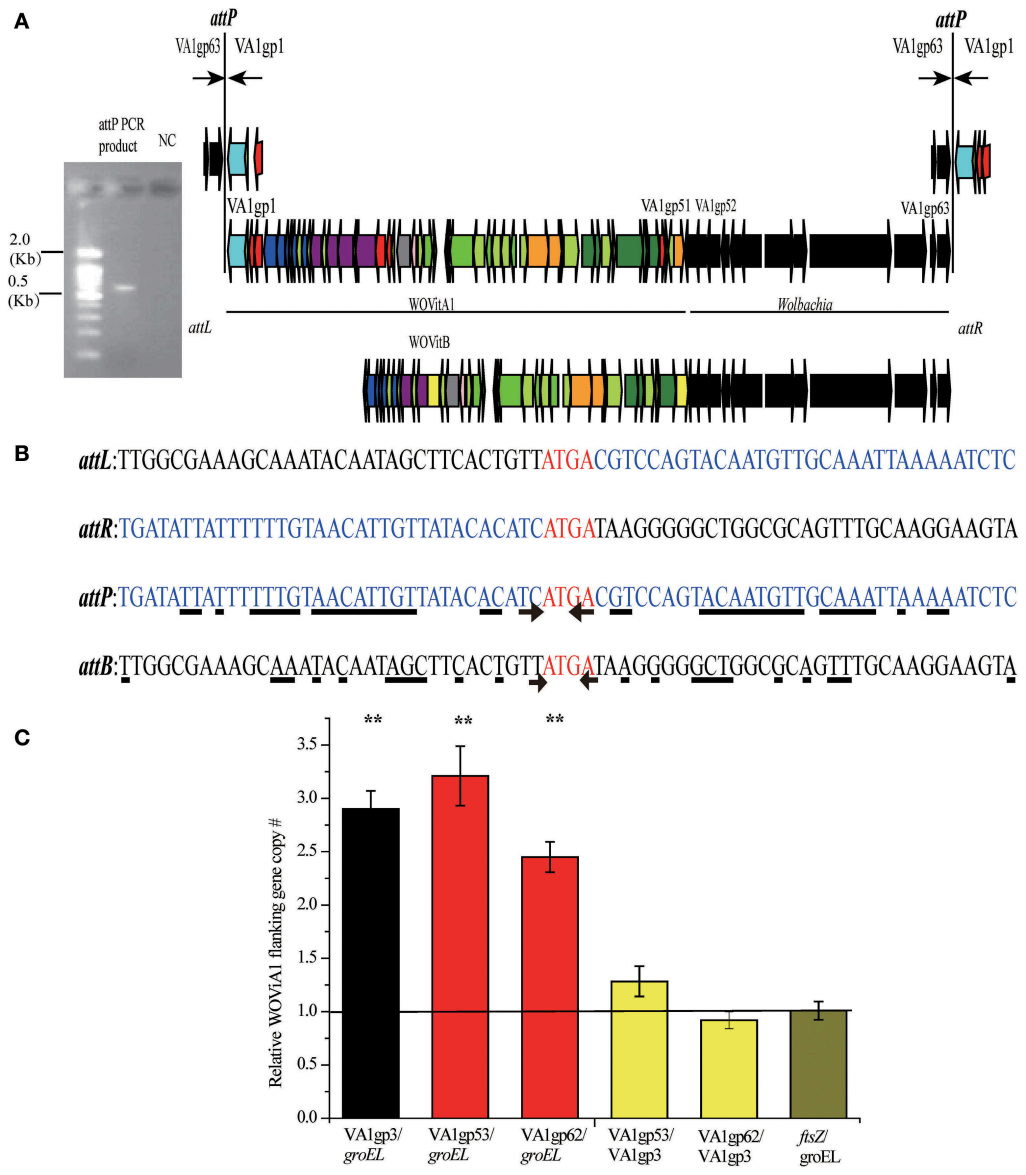
**Determination of WOVitA1 prophage region. (A)** *attP* PCR product, a 0.6 kb PCR product containing the WOVitA1 *attP* region. NC, negative control with distilled water as template. Black arrows show the locations of outward primers. Genes are presented by arrows while psudogenes and non-coding regions are boxes. Colors of ORFs are as described in the legend of Figure 2. **(B)** Alignment of the *attP* PCR product sequence and the *w*VitA sequence. The arrow indicates the beginning of the inverted repeat sequences, the underline shows nucleotide which we can find the corresponding inverted repeat sequences beside the core sequence. **(C)** Relative copy number of defined prophage WOVitA1 region, prophage WOVitA1 flanking region, and *w*VitA of *N. vitripennis*. Relative copy number of ORFs encoding genes *groEL and ftsz* represented *w*VitA, VA1gp3 represented prophage WOVitA1, and VA1gp53 and VA1gp62 represented WOVitA1 flanking region were measured by real-time qPCR. The black line (number one) depicts the expected copy number. Error bars represent one standard deviation. The double asterisk indicates a significant difference (*P* < 0.01; two-tailed *t*-test).

Homologs of two *ank* genes from WOcauB2, B2gp46, and B2gp47, are present in six of the 32 sequenced *Wolbachia* strains (Table 2). Homologs of B2gp46 are present in *Wolbachia* strains *w*Ri, *w*VitA, and *w*VitB from *N. vitripennis* (Kent et al., 2011b), *w*No from *D. simulans* (Ellegaard et al., 2013), *w*AlbB from *Aedes albopictus* (Mavingui et al., 2012), and *w*Ana from *Drosophila ananassae* (Salzberg et al., 2005). B2gp47 has homologs in *Wolbachia* strains *w*Ri, *w*VitA, and *w*VitB. These scattered distribution patterns indicate recent transmission of the two *ank* genes. The segmentation point by cumulative GC profile of phage WOcauB2 supports that the phage WOcauB2 has acquired the two *ank* genes, B2gp46 and B2gp47, from a foreign DNA source (Figure S3). Furthermore, both genes are located at the 3′ ends of phages WOcauB2, WORiC, WOVitA1, and WOVitB (Figures 4A, 5) and are packaged into WOcauB2 (Tanaka et al., 2009) and WOVitA1 particles (see following part a), indicating that phage WO is the vehicle of their transmission.

**Table 2:** **Distribution of B2gp46 and B2gp47 genes in WOcauB2 with highly similar positional homologs in other sequenced ***Wolbachia*** genomes**.

***Wolbachia***	**Homolog to B2gp46**	**Homolog to B2gp47**
*w*Ri	WRi_006870 (98%)	WRi_006860 (99%)
wVitA	VA1gp58 (98%)	VA1gp59 (98%)
wVitB	WOVitB45 (98%)	WOVitB46 (98%)
wNo	wNo_02110 (98%)	–
	wNo_10630^p^ (81%)	
wAlbB	WALBB_550005 (98%)	–
*w*Ana	WwAna0563^a^ (99%)	–

Stringency parameters for blast search: >40% coverage, >40% identity. Numbers in parentheses indicate the sequence similarities between homologs.

p Pseudogene.

a Partial sequences, located at contig ends.

–, no information.

### Phage WOVitA1 mediates the transfer of the flanking region

In addition to WOcauB2 and WOcauB3, *Wolbachia w*VitA possesses another active phage, WOVitA1, which has been transferred to *Wolbachia w*VitB in *N. vitripennis* (the phage WOVitA1 in *w*VitB is WOVitB1; Kent et al., 2011b). It is interesting to note that both WOVitA1 and WOVitB not only have nearly identical phage regions (the WOVitA1 region is VA1gp1–VA1gp51) but also highly similar flanking regions with bacterial genes (VA1gp52–VA1gp63 in WOVitA1; Kent et al., 2011b). Here, by analyzing the attachment site (*att*) of WO phages (*attP*) and the density correlation of WO with its *Wolbachia* host, we demonstrate that the active phage WOVitA1 can mediate the transfer of its flanking bacterial region.

#### The VA1gp52–VA1gp63 flanking region is packaged into active WOVitA1 particles

The core sequence of the attachment site is the region where the phage undergoes site-specific recombination, which occurs when the phage integrates into and excises out of the bacterial host genome (Smith and Thorpe, 2002). Phage WO has a self-ligated circular genome (Tanaka et al., 2009). If the flanking *Wolbachia* genes, VA1gp52–VA1gp63, are included in phage WOVitA1 particles, PCR with outward primers at the end of the prophage WOVitA1 and the flanking region would be expected to yield an *attP* site product. Indeed, we obtain the *attP* site product (Figure 5A) by using these primers (Table S3). By comparing the *attP, attB* (bacterial *att* site), *attL* (left prophage *att* site), and *attR* (right prophage *att* site) sequences, we discovered that the tetranucleotides ATGA are identical among the *att* sites (Figure 5B). Thus, these sequences are inferred to be the candidate core sequence for WOVitA1. However, for phages WOcauB2 and WOcauB3, the core sequences are only a single nucleotide T and trinucleotides TTG, respectively (Tanaka et al., 2009). Further, the core sequence of WOVitA1 is flanked by a pair of inverted repeat sequences (Figure 5B), where the core sequences of WOcauB2 and WOcauB3 are not (Tanaka et al., 2009).

To assess relative copy number of phage and *Wolbachia*, we measured the copy number of the *w*VitA genome (represented by both the single-copy heat-shock protein 60 gene *groEL* and the cell division gene *ftsZ*), the phage WOVitA1 genome (represented by the single-copy gene *ank*, VA1gp3), and the phage WOVitA1 flanking region (represented by both the single-copy transcriptional regulator gene VA1gp53 and the Hsp20-family heat shock protein gene VA1gp62; the primers are listed in Table S3). With a single lysogenic copy of WOVitA1, the WOVitA1 density should always equal (no lytic activity) or exceed (with lytic activity producing multiple phage virions) the *w*VitA copy number. Additionally, if the region flanking WOVitA1 is packaged into the virion, it should also exceed the genome copy number during lytic replication. The phage to *Wolbachia* ratio was determined to be 2.90 ± 0.17 for VA1gp3: *groEL* (*p* < 0.01; two-tailed *t*-test), while the ratios of the phage flanking region to *Wolbachia* were measured as 3.21 ± 0.28 for VA1gp53: *groEL* and 2.45 ± 0.14 for VA1gp62: *groEL* (all *p* < 0.01; two-tailed *t*-test; Figure 5C). For comparison, the phage flanking region to phage ratios were 1.28 ± 0.14 for VA1gp53: VA1gp3 and 0.92 ± 0.08 for VA1gp62: VA1gp3 and the *Wolbachia* to *Wolbachia* ratio is 1.01 ± 0.09 for *ftsZ: groEL*. These results indicate that the flanking region is part of WOVitA1 and is being replicated extrachromosomally. In addition, in the cumulative GC profile of phage WOVitA1, the segmentation point includes VA1gp52–VA1gp63, further indicating that the phage WO acquired the region from foreign DNA sources (Figure S4). Thus, we propose that *Wolbachia* genes VA1gp52–VA1gp63 were transmitted along with the transmission of the phage WOVitA1 (VA1gp1–VA1gp51) to *w*VitB (Kent et al., 2011b).

#### The potential roles of packaged genes in phage WOVitA1 particles

Because most of the packaged *Wolbachia* genes (VA1gp52–VA1gp63) are conserved among many bacteria (Kent et al., 2011b), we can predict their functions using blastp search and further trace their origins. The three genes VA1gp52, VA1gp53, and VA1gp56 are transcriptional regulators homologous to *wtrM* in *w*PipMol, which is implicated in cytoplasmic incompatibility (CI) in *Culex* mosquitoes via regulating mosquito gene expression (Pinto et al., 2013). The packaged genes may also encode DNA repair protein RadC (VA1gp55), adaptor protein MutL (VA1gp57), heat shock protein (VA1gp62), and ANK proteins (VA1gp58, VA1gp59, VA1gp60, and VA1gp61). All of these genes function in DNA binding or protein-protein interactions and could be involved in CI (Penz et al., 2012).

### Inactive phages WO are transferred and mediate gene transfer

**Figure 6:**
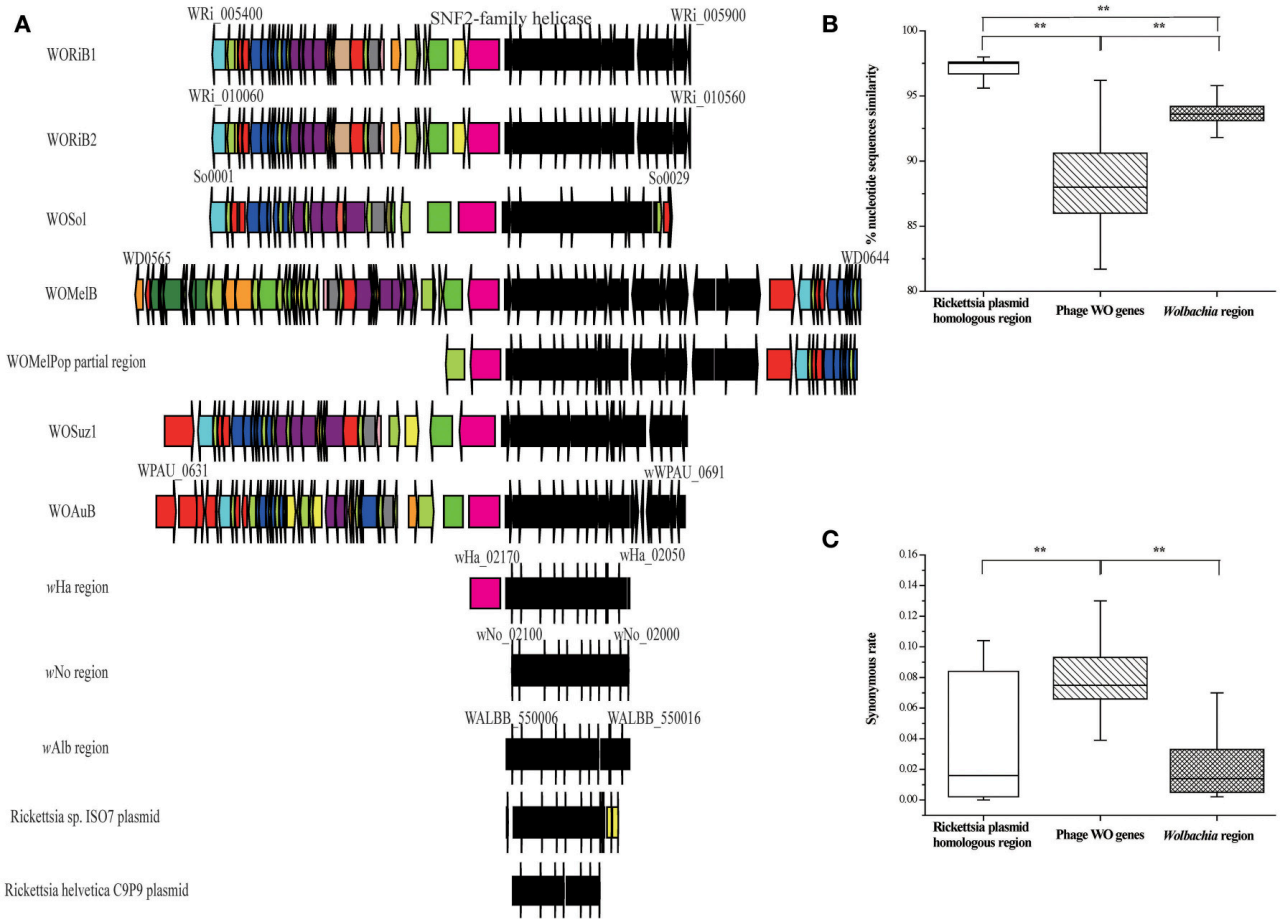
**A conserved bacterial region associated with phage WO, ***Wolbachia*** strains, and ***Rickettsia*** plasmid. (A)** Structural comparisons of the gene compositions among prophage WO, *Wolbachia*, and plasmid of *Rickettsia* endosymbiont in *Ixodes scapularis* and *I. ricinus*, all of which have the same conserved bacterial region genes (WRi_005730–WRi_005830) with WORiB1 flanking region. Genes are presented by arrows while psudogenes and non-coding regions are boxes. Colors of ORFs are as described in the legend of Figure 2. **(B)** Percent nucleotide identity between genes from the conserved bacterial region, phage WO genes (WORiB1, WORiB2, WOSol, WOMelB, WOSuz1, and WOAuB), and *Wolbachia* genes. **(C)** Synonymous rate between genes from the conserved bacterial region, phage WO genes (WORiB1, WORiB2, WOSol, WOMelB, WOSuz1, and WOAuB), and *Wolbachia* genes. Error bars represent one standard deviation. The double asterisk indicates a significant difference (*P* < 0.01; Mann–Whitney U, two-tailed test).

Inactive WO phages may also have been involved in gene transfer events. In eight *Wolbachia* genomes, we detected a conserved bacterial region extending over 20 kb that is highly homologous (>70% nt identity) to regions in the bacterial plasmids of *Rickettsia buchneri* sp. nov. and *Rickettsia helvetica* (Ishmael et al., 2009). These bacteria infect *Ixodes scapularis* (Kurtti et al., 2015) and *Ixodes ricinus* (Dong et al., 2012) ticks respectively. Interestingly, except in *w*No and *w*Alb, this region in each of the other *Wolbachia* genomes is inserted in or near the phage WO, and some of the associated WO phages are degenerate (Figure 6A). We also detect homologs of some of the genes from this region in *w*Bol1 (from *Hypolimnas bolina*; Duplouy et al., 2013), *w*VitB, *w*Wil (from *Drosophila willistoni*) (Craig Venter Institute), *w*Coc (from *Dactylopius coccus*; Campana et al., 2015), and *w*Rec (from *Drosophila recens*; Metcalf et al., 2014). However, all of the homologs are in scaffolds with small sizes, which prevents us from obtaining their flanking regions; therefore, we did not further analyze them. In public databases, there are no closely related orthologs of this bacterial region except in the *Wolbachia* strains and the two *Rickettsia* strains as mentioned above. There are at least three possible explanations for the distribution pattern of this region. First, this bacterial region is one of the modules of phage WO. Second, this conserved bacterial region has been frequently and independently inserted into *Wolbachia* at the same phage WO location, where there exists an active cloning location. Third, phage WO or plasmid mediates the transfer of this bacterial region among different *Wolbachia* strains, between *Rickettsia* from *I. scapularis* and *I. ricinus*, or between the two bacterial genera. However, the average nucleotide identity of the genes in this region (97.00%) is significantly higher than that of the other *Wolbachia* genes (93.28%; MWU, two-tailed, *p* < 0.01; Figure 6B and Table S4) and is also significantly higher than that of WORiB1, WORiB2, WOSol, WOMelB, WOSuz1, and WOAuB (88.67%; MWU, two-tailed, *p* < 0.01; Figure 6B). Furthermore, the synonymous mutation rate of the genes in the region from *w*Ri, *w*Cs, *w*Mel, *w*Suz, and *w*Au (0.02) is significantly lower than the average synonymous mutation rate of the associated phage WO genes from WORiB1, WORiB2, WOSol, WOMelB, WOSuz1, and WOAuB (0.08; MWU, two-tailed, *p* < 0.01; Figure 6C). The average nucleotide identity and synonymous mutation rate analyses indicate this bacterial region is not phage WO module. Additionally, in the cumulative GC profile of phage WOMelB (Figure S5), the segmentation point, including the putative HGTs, also demonstrates phage WO and this bacterial region are from different DNA sources. Based on these analyses, we exclude the first explanation. However, we cannot exclude the second possibility that this bacterial region has been frequently and independently inserted into *Wolbachia* at the same phage WO location. Considering that phage and plasmid are two of the most common genetic vectors in nature (Syvanen, 1994; Canchaya et al., 2003), it is thus a more parsimonious explanation that phage WO or *Rickettsia* plasmid may have recently mediated the transfer of these genes.

Within the transfered region, there are 11 conserved genes (WRi_005730–WRi_005830; Figure 6A). These genes encode an NAD-dependent epimerase/dehydratase family protein, a glycosyltransferase, two putative L-allo-threonine aldolases, an ABC transporter permease, a GlpT/PgpT/UhpT transporter family protein, a UDP-glucose 6-dehydrogenase, and three conserved hypothetical proteins. Many of these proteins play a role in the synthesis and degradation of surface polysaccharides, which could alter the ability of different *Wolbachia* strains to interact with eukaryotic hosts (Ishmael et al., 2009).

Moreover, this transfered region is located adjacent to a conserved gene encoding a SNF2-family helicase, which was detected in a phage region via genome analysis (Ishmael et al., 2009). In eukaryotes, this gene may function in processes including transcriptional regulation, the maintenance of chromosome stability during mitosis and the processing of DNA damage (Eisen et al., 1995).

## Discussion

### Effect of transferred genes on the host

Transfer events mediated by phage WO can shape the genome composition of *Wolbachia*. For example, ANKs are rare in bacteria but common in eukaryotes and viruses (Bork, 1993; Li et al., 2006), while these genes are overrepresented in *Wolbachia* bacteria. For example, there are typically only 1–3 *ank* genes in the α-Proteobacteria (Andersson et al., 1998; Caturegli et al., 2000), but there are 60 *ank* genes in *Wolbachia w*Pip from *C. pipiens* (the largest number of *ank* genes in any sequenced bacterial genome; Klasson et al., 2008), 35 in *w*Ri (Klasson et al., 2009), and 23 in *w*Mel (Wu et al., 2004). In this study, we demonstrate that WO phage particles can package and mediate the transfer of “extra” *ank* genes into *Wolbachia* genomes, e.g., B2gp46 and B2gp47 in WOcauB2, both of which are of non-*Wolbachia* origin. Moreover, WO can also mediate the transfer of “extra” *ank* genes between different *Wolbachia* strains. These results indicate that the WO-mediated transfer of “extra” *ank* genes may be a partial explanation for the abundance of *ank* genes in *Wolbachia* compared to other closely related bacteria.

The discovery of these horizontal transfer events raises the question of whether transferred genes play a role in *Wolbachia* or their eukaryotic hosts. Prophage-encoded virulence factors are important for a number of bacterial species, and these genes can increase pathogenicity or result in the emergence of new pathogens (Canchaya et al., 2003; Brüssow et al., 2004). This phenomenon has been recognized for the toxins of *Vibrio cholerae* (Waldor and Mekalanos, 1996), *Streptococcus pyogenes* (Broudy et al., 2002), and *Hamiltonella defensa* (Moran et al., 2005; Oliver et al., 2009), all of which are phage-encoded. Here, the presence of the transferred *spvB* motif gene in phage WO particles, and the role of this gene in WO infection of *Wolbachia* and the corresponding eukaryotic hosts needs further study.

*Wolbachia* mediated mosquito-borne disease control is a hot topic (Dobson et al., 2016; Loreto and Wallau, 2016; O'Neill, 2016; Waltz, 2016). Caged and open-field experiments showed that the *w*Mel *Wolbachia* strain is able to block dengue transmission (Walker et al., 2011). However, there is also a potential risk that the *Wolbachia* strains, along with phage WO and other genes, may be transferred to other insects (Loreto and Wallau, 2016). Here we demonstate that phage WO can mediate HGT among different *Wolbachia* strains. Thus, future studies should also evaluate the biosafety of this phage vector when utilizing *Wolbachia*-infected mosquitos.

### Phage WO has the potential to be reengineered as a transformation tool for *wolbachia*

The phenomenon of eukaryotic host reproductive manipulation by *Wolbachia* is compelling, but the underlying mechanism still remains poorly characterized due to the lack of robust tools for transforming *Wolbachia* (Werren, 1997; LePage and Bordenstein, 2013). The phage WO has been proposed as the only potential transformation tool for *Wolbachia* (Fujii et al., 2004; Metcalf and Bordenstein, 2012). However, it remains unclear whether phage WO can be successfully used as such a tool, and there is little research concerning this issue (LePage and Bordenstein, 2013). Here, we show that phage WO can mediate gene transfer; the active phage WOVitA1 has typical characteristics of the core sequences. Additionally, the 3′ end of WO prophages sites might be used as multiple-cloning sites. All of these results further support that phage WO has the potential to be utilized as a genetic vector for the study of *Wolbachia*.

### Phage WO let us rethink endosymbiont genome evolution theory

In the evolution of intracellular endosymbionts, genome reduction is the predominant trend differentiating endosymbionts from free-living bacteria. Additionally, intracellular endosymbionts are strictly constrained to living inside host-derived cells: their effective population size is reduced, which renders selection less efficient; they have limited opportunities to come into contact with other unrelated bacteria and have little chance to exchange genetic material; the stable and rich nutrients of the intracellular environment remove selection constraints on genes (like mobile DNA) that are no longer strictly required (Bordenstein and Reznikoff, 2005; Moya et al., 2008; Moran and Bennett, 2014). However, phage WO is widespread among *Wolbachia* genomes (present in about 89%; Bordenstein and Wernegreen, 2004) and even can comprise more than 20% of mobile DNA genes in *Wolbachia* (Chafee et al., 2010). What's more, the active mobile elements located within the genomes of endosymbionts can still mediate the deletion and insertion of genetic components at different locations in the genome. Based on several lines of evidence, the present study shows that the phage WO could mediate HGT between different *Wolbachia* strains of genes from *Wolbachia* and unrelated bacterial lineages, which showes *Wolbachia* genomes are not stable and might gain new genes by phage WO.

## Author contributions

GW, conception and design, acquisition of data, analysis, and interpretation of data, drafting and revising the article; DH, conception and design; JX, analysis and interpretation of data, drafting the article; BS and TX, analysis and interpretation of data; KM, analysis and drafting the article; YW, acquisition of data.

### Conflict of interest statement

The authors declare that the research was conducted in the absence of any commercial or financial relationships that could be construed as a potential conflict of interest.

## Supplementary material

Table S1 **Nucleotide identity of Wolbachia protein-coding genes between wCauB and wNo**.

Table S2 **Nucleotide identity of Wolbachia protein-coding genes between wCauB and wRi**.

Table S3 **Description of primers used in study**.

Table S4 **Selected Wolbachia genes in the wRi genome and their homologs in other Wolbachia genomes**.

Figure S1 **Structural comparison between prophage WO. (A)** Gene order comparisons among phage WOcauB3, WONo1, and WONo3 (WONo2 is too short and we didn't take it for further analysis), **(B)** Gene order comparisons among phage WOcauB2, WORiA, and WORiB1 (WORiB1 and WORiB2 are two identical copies and we just took WORiB1 for analysis). Gray lines connect matched ORFs with *E* < 1e−15. Colors of ORFs are as described in the legend of Figure 2.

Figure S2 **Phylogenetic analysis of ***Wolbachia*** MLST genes**. Maximum likelihood phylogenetic analyse demonstrates *w*No and *w*CauB, *w*Ri, and *w*CauB are divergent *Wolbachia* strains. The name of each sequence is the abbreviation of the *Wolbachia* strain (Table 1). Capital letters indicate *Wolbachia* strain supergroup affiliation from the literature. MLST: multi-locus sequence typing (with genes of *coxA, fbpA, ftsZ, gatB*, and *hcpA*).

Figure S3 **The cumulative GC profile for prophage WOcauB2. (A)** Gene presence in prophage WOcauB2. Colors of ORFs are as described in the legend of Figure 2. **(B1)** z′ curve for prophage WOcauB2. Segmentation points are marked with green squares. Segmentation point coincides with the HGT (B2gp46–B2gp47). **(B2)** The GC content distribution of prophage WOcauB2, using a 100 bp sliding window.

Figure S4 **The cumulative GC profile for prophage WOVitA1. (A)** Gene presence in prophage WOVitA1. Colors of ORFs are as described in the legend of Figure 2. **(B1)** z′ curve for prophage WOVitA1. Segmentation points are marked with green squares. Segmentation point includes the HGT (VA1gp52–VA1gp63). **(B2)** The GC content distribution of prophage WOVitA1, using a 100 bp sliding window.

Figure S5 **The cumulative GC profile for prophage WOMelB. (A)** Gene presence in prophage WOMelB. Colors of ORFs are as described in the legend of Figure 2. **(B1)** z′ curve for prophage WOMelB. Segmentation points are marked with green squares. Segmentation point includes the HGT (WD0611–WD0632). **(B2)** The GC content distribution prophage WOMelB, using a 100 bp sliding window.
