# Supplementary material for: Bacteriophage WO Can Mediate Horizontal Gene Transfer in Endosymbiotic Wolbachia Genomes
Source: Front Microbiol. 2016 Nov 29;7:1867. doi: 10.3389/fmicb.2016.01867 (PMC5126046; doi:10.3389/fmicb.2016.01867)
Supplement: Table S1 — Nucleotide identity of Wolbachia protein-coding genes between wCauB and wNo. [file Table1.DOCX]

**Table S1 Nucleotide identity of *Wolbachia* protein-coding genes between *w*CauB and *w*No.**

| **Gene** | **% identity** | **alignment length (bp)** | | **E value** |
| --- | --- | --- | --- | --- |
| GF1gp1 | 94.58 | | 369 | 1.00E-165 |
| GF1gp2 | 99.28 | | 558 | 0 |
| GF1gp3 | 93.28 | | 119 | 3.00E-46 |
| GF1gp4 | 95.24 | | 609 | 0 |
| GF1gp5 | 96.94 | | 1274 | 0 |
| GF1gp6 | 96.23 | | 318 | 8.00E-149 |
| GF1gp7 | 94.39 | | 1212 | 0 |
| GF1gp8 | 92.61 | | 501 | 0 |
| GF1gp9 | 92.60 | | 1540 | 0 |
| GF1gp11 | 97.41 | | 771 | 0 |
| GF1gp12 | 99.39 | | 978 | 0 |
| GF1gp13 | 99.53 | | 426 | 0 |
| GF1gp14 | 99.24 | | 792 | 0 |
| GF1gp15 | 99.47 | | 1137 | 0 |
| GF1gp16 | 99.49 | | 789 | 0 |
| GF1gp17 | 98.91 | | 459 | 0 |
| GF1gp19 | 97.71 | | 1137 | 0 |
| GF1gp20 | 86.40 | | 375 | 2.00E-126 |
| GF1gp21 | 96.77 | | 1239 | 0 |
| GF2gp1 | 95.10 | | 429 | 0 |
| GF2gp2 | 91.19 | | 386 | 4.00E-149 |
| GF2gp3 | 83.18 | | 220 | 4.00E-50 |
| GF2gp4 | 94.82 | | 1391 | 0 |
| GF2gp5 | 92.75 | | 579 | 0 |
| GF2gp6 | 89.54 | | 1186 | 0 |
| GF2gp7 | 94.03 | | 1324 | 0 |
| GF2gp9 | 91.78 | | 645 | 0 |
| GF2gp10 | 99.31 | | 1443 | 0 |
| GF2gp12 | 99.46 | | 1488 | 0 |
| GF2gp15 | 92.49 | | 852 | 0 |
| GF2gp16 | 97.96 | | 588 | 0 |
| GF2gp21 | 90.41 | | 949 | 0 |
| GF2gp22 | 93.91 | | 1083 | 0 |
| GF2gp26 | 97.45 | | 345 | 2.00E-142 |
| GF2gp27 | 98.21 | | 894 | 0 |
| GF2gp28 | 98.71 | | 855 | 0 |
