# Supplementary material for: Bacteriophage WO Can Mediate Horizontal Gene Transfer in Endosymbiotic Wolbachia Genomes
Source: Front Microbiol. 2016 Nov 29;7:1867. doi: 10.3389/fmicb.2016.01867 (PMC5126046; doi:10.3389/fmicb.2016.01867)
Supplement: Table S2 — Nucleotide identity of Wolbachia protein-coding genes between wCauB and wRi. [file Table2.DOCX]

**Table S2 Nucleotide identity of *Wolbachia* protein-coding genes between *w*CauB and *w*Ri.**

| **Gene** | **% identity** | **alignment length (bp)** | | **E value** |
| --- | --- | --- | --- | --- |
| GF1gp1 | 81.96 | | 327 | 1.00E-89 |
| GF1gp2 | 86.74 | | 558 | 0 |
| GF1gp4 | 85.76 | | 618 | 0 |
| GF1gp5 | 86.9 | | 1267 | 0 |
| GF1gp7 | 90.61 | | 1182 | 0 |
| GF1gp8 | 90.42 | | 501 | 0 |
| GF1gp9 | 90.40 | | 1394 | 0 |
| GF1gp11 | 86.67 | | 771 | 0 |
| GF1gp12 | 86.38 | | 983 | 0 |
| GF1gp13 | 86.04 | | 426 | 7.00E-145 |
| GF1gp14 | 85.84 | | 781 | 0 |
| GF1gp15 | 89.45 | | 1137 | 0 |
| GF1gp16 | 84.19 | | 787 | 0 |
| GF1gp17 | 86.48 | | 430 | 2.00E-133 |
| GF1gp18 | 87.15 | | 392 | 6.00E-133 |
| GF1gp19 | 89.31 | | 1144 | 0 |
| GF1gp21 | 79.80 | | 1225 | 0 |
| GF1gp22 | 98.71 | | 2169 | 0 |
| GF2gp1 | 83.92 | | 429 | 3.00E-131 |
| GF2gp2 | 81.65 | | 387 | 3.00E-106 |
| GF2gp3 | 68.26 | | 482 | 8.00E-43 |
| GF2gp4 | 87.73 | | 1402 | 0 |
| GF2gp5 | 79.69 | | 586 | 1.00E-149 |
| GF2gp6 | 87.82 | | 1174 | 0 |
| GF2gp7 | 90.56 | | 1335 | 0 |
| GF2gp8 | 77.78 | | 216 | 1.00E-43 |
| GF2gp9 | 82.61 | | 644 | 0 |
| GF2gp10 | 88.24 | | 1446 | 0 |
| GF2gp12 | 90.26 | | 1489 | 0 |
| GF2gp13 | 83.67 | | 649 | 0 |
| GF2gp14 | 89.70 | | 738 | 0 |
| GF2gp15 | 85.80 | | 859 | 0 |
| GF2gp16 | 85.88 | | 588 | 0 |
| GF2gp19 | 90.00 | | 370 | 1.00E-142 |
| GF2gp20 | 90.35 | | 342 | 6.00E-132 |
| GF2gp21 | 89.63 | | 945 | 0 |
| GF2gp21 | 93.26 | | 1083 | 0 |
| GF2gp23 | 89.86 | | 355 | 3.00E-136 |
| GF2gp24 | 90.99 | | 333 | 2.00E-132 |
| GF2gp25 | 95.45 | | 660 | 0 |
| GF2gp26 | 87.69 | | 471 | 3.00E-169 |
| GF2gp27 | 88.26 | | 869 | 0 |
| GF2gp28 | 79.50 | | 800 | 0 |
