# Supplementary material for: Bacteriophage WO Can Mediate Horizontal Gene Transfer in Endosymbiotic Wolbachia Genomes
Source: Front Microbiol. 2016 Nov 29;7:1867. doi: 10.3389/fmicb.2016.01867 (PMC5126046; doi:10.3389/fmicb.2016.01867)
Supplement: Table S3 — Description of primers used in study. [file Table3.DOC]

**Table S3 Description of primers used in study.**

| Gene | Forward primer (5’-3’) | Reverse primer (5’-3’) | | Amplicon length (bp) | Reference |
| --- | --- | --- | --- | --- | --- |
| *attP* | TGAAAGCCTAAGACCACC | | AAGTAAGGAAGAATTTAAACAG | 600 | this study |
| *groEL* | CAACCTTTACTTCCTATTCTTG | | CTAAAGTGCTTAATGCTTCACCTTC | 97 |  |
| *ftsZ* | CGTGGATACACTTATTGTCATTC | | TTGCCCATCTCGCTCAT | 198 | this study |
| *VA1gp3* | TCGCATAATCTATTGCTGTC | | GCGGTAACGGAGAAACGT | 147 | this study |
| *VA1gp53* | TATCCTTTGCCCCACTTTGTATG | | AAGGGGTTTGATGAAAGTAGGAGTT | 118 | this study |
| *VA1gp62* | ATAGTGAAGGGTGAAAAGAC | | TGCTCTACATTTACTGGAAG | 116 | this study |

**References**

1. **Bordenstein SR, Marshall ML, Fry AJ, Kim U, Wernegreen JJ.** 2006. The tripartite associations between bacteriophage, *Wolbachia*, and arthropods. PLoS Pathog. **2:**e43.
