# Supplementary material for: Bacteriophage WO Can Mediate Horizontal Gene Transfer in Endosymbiotic Wolbachia Genomes
Source: Front Microbiol. 2016 Nov 29;7:1867. doi: 10.3389/fmicb.2016.01867 (PMC5126046; doi:10.3389/fmicb.2016.01867)
Supplement: Table S4 — Selected Wolbachia genes in the wRi genome and their homologs in other Wolbachia genomes. [file Table4.DOC]

**Table S4 Selected *Wolbachia* genes in the *w*Ri genome and their homologs in other *Wolbachia* genomes.**

| *Wolbachia*  Gene | *w*Mel | *w*Ha | *w*No | *w*Cs | *w*MelPop | *w*AlbB | *w*lb_suzi | *w*Au |
| --- | --- | --- | --- | --- | --- | --- | --- | --- |
| WRi_001070 | WD_0146 | wHa_00620 | wNo_00350 | KP966834 | AQQE01000016 | CAGB01000095 | CAOU02000004 | CDR78488.1_86 |
| WRi_007520 | WD_0723 | wHa_06140 | wNo_02790 | KP966835 | AQQE01000047 | CAGB01000132 | CAOU02000026 | CDR79102.1_700 |
| WRi_002620 | WD_0484 | wHa_02960 | wNo_08660 | KP966836 | AQQE01000033 | CAGB01000012 | CAOU02000009 | CDR78904.1_502 |
| WRi_000100 | WD_0009 | wHa_00090 | wNo_10770 | KP966838 | AQQE01000006 | CAGB01000014 | CAOU02000001 | CDR78411.1_9 |
| WRi_011190 | WD_1085 | wHa_09060 | wNo_07610 | KP966832 | AQQE01000064 | CAGB01000124 | CAOU02000036 | CDR79405.1_1003 |
| WRi_011430 | WD_1167 | wNo_10320 | wNo_04930 | KP966833 | AQQE01000002 | CAGB01000118 | CAOU02000037 | CDR79526.1_1124 |
| WRi_004870 | WD0650 | wHa_05470 | wNo_08540 | KP966837 | AQQE01000044 | CAGB01000009 | CAOU02000007 | CDR78957.1_555 |
| WRi_007310 | WD0068 | wHa_06330 | wNo_03720 | wSo0001 | AQQE01000047 | CAGB01000143 | CAOU02000026 | CDR79124.1_722 |
| WRi_003480 | WD_0534 | wHa_0428 | wNo_08900 | KP966839 | AQQE01000030 | CAGB01000014 | CAOU02000011 | CDR78937.1_535 |
| WRi_002400 | WD_0251 | wHa_02000 | wNo_10500 | wSo0029 | AQQE01000022 | CAGB01000148 | CAOU02000008 | CDR78626.1_224 |
| WRi_008730 | WD0914 | wHa_07730 | wNo_11130 | KP966840 | AQQE01000056 | CAGB01000019 | CAOU02000030 | CDR79243.1_841 |
