# Supplementary figures and images for: Bacteriophage WO Can Mediate Horizontal Gene Transfer in Endosymbiotic Wolbachia Genomes
Source: Front Microbiol. 2016 Nov 29;7:1867. doi: 10.3389/fmicb.2016.01867 (PMC5126046; doi:10.3389/fmicb.2016.01867)

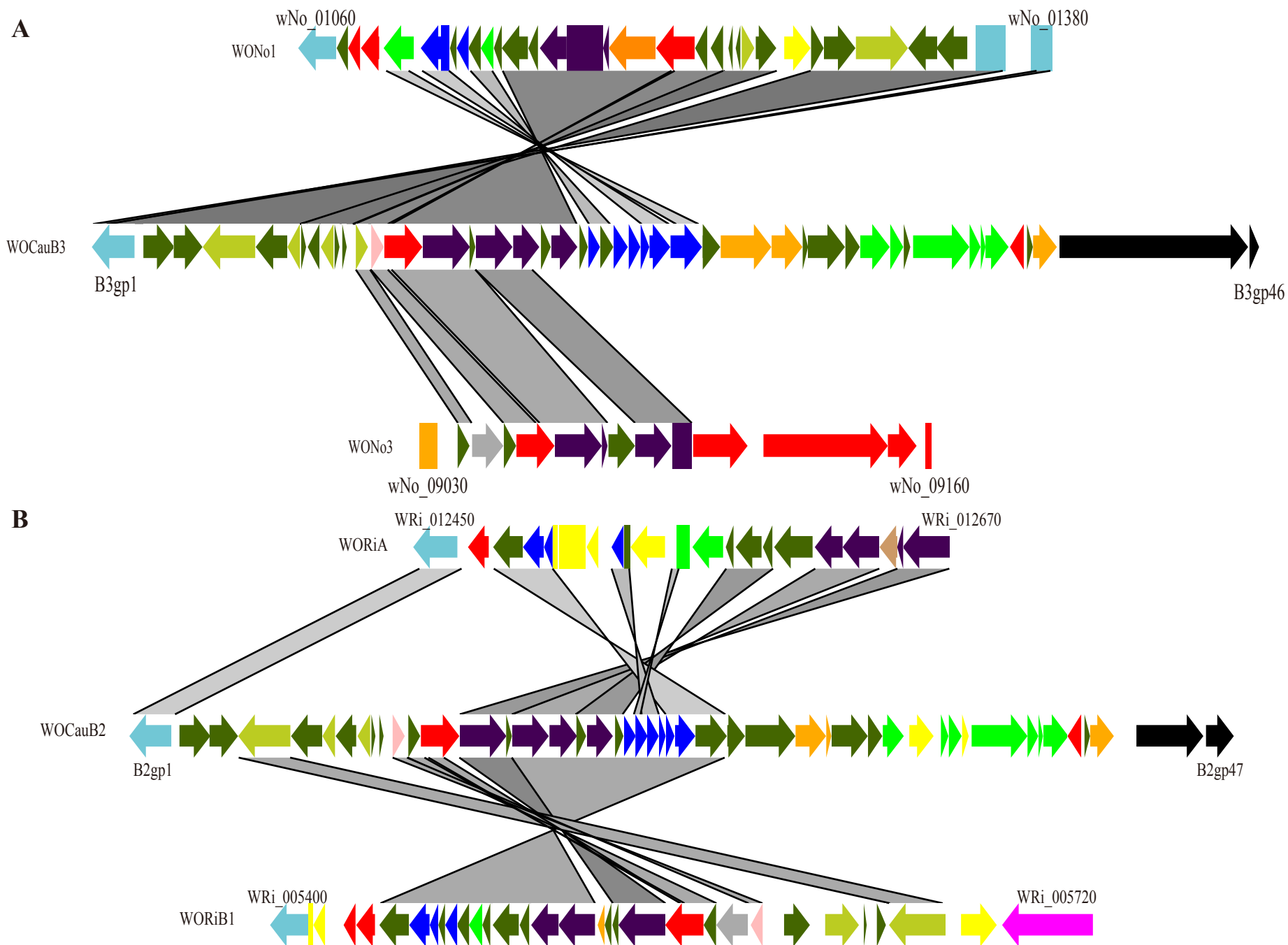

Supplement: Figure S1 — Structural comparison between prophage WO. (A) Gene order comparisons among phage WOcauB3, WONo1, and WONo3 (WONo2 is too short and we didn't take it for further analysis), (B) Gene order comparisons among phage WOcauB2, WORiA, and WORiB1 (WORiB1 and WORiB2 are two identical copies and we just took WORiB1 for analysis). Gray lines connect matched ORFs with E < 1e−15. Colors of ORFs are as described in the legend of Figure 2. [file Image1.PDF]

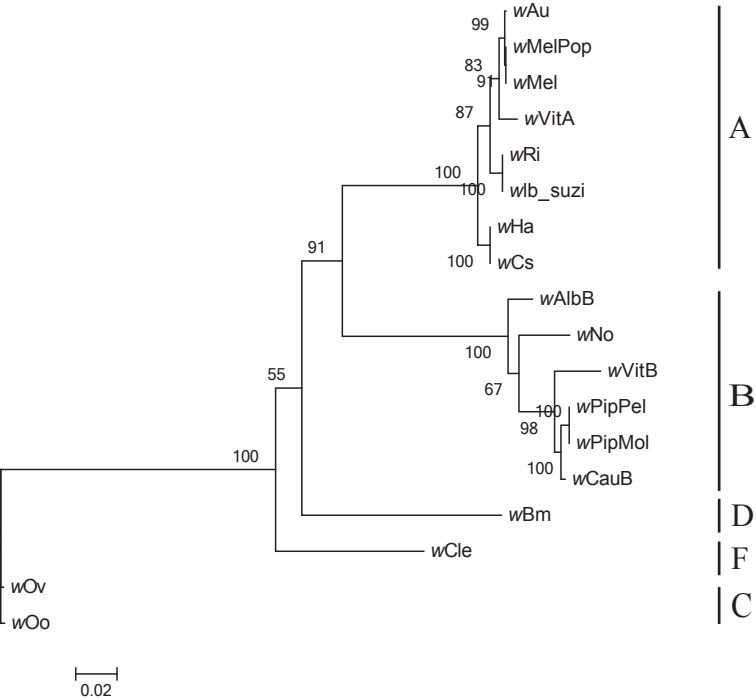

Supplement: Figure S2 — Phylogenetic analysis of Wolbachia MLST genes. Maximum likelihood phylogenetic analyse demonstrates wNo and wCauB, wRi, and wCauB are divergent Wolbachia strains. The name of each sequence is the abbreviation of the Wolbachia strain (Table 1). Capital letters indicate Wolbachia strain supergroup affiliation from the literature. MLST: multi-locus sequence typing (with genes of coxA, fbpA, ftsZ, gatB, and hcpA). [file Image2.PDF]

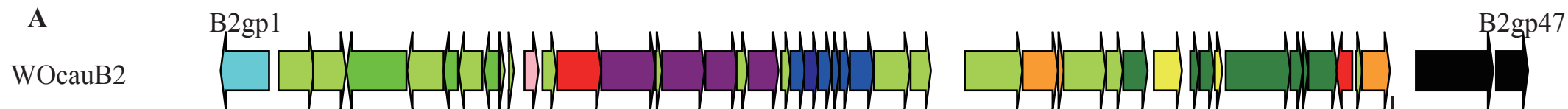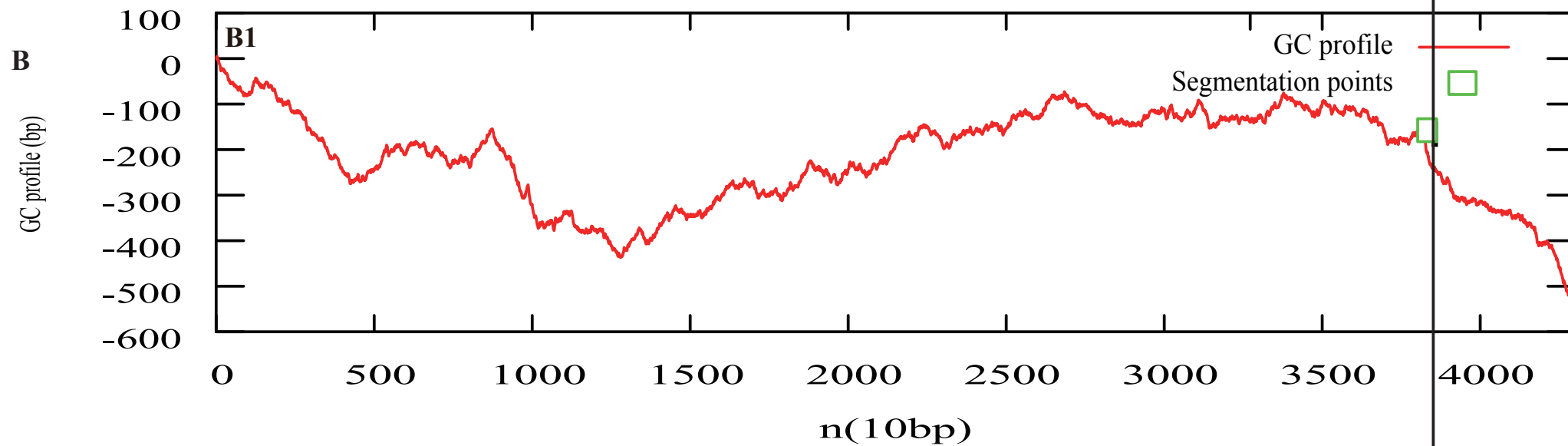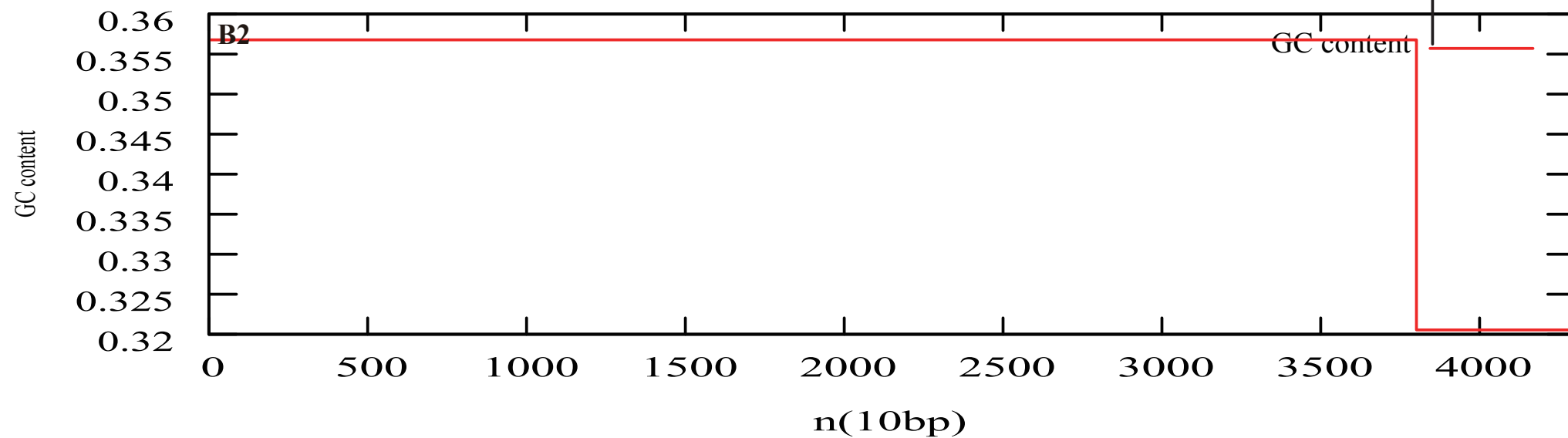

Supplement: Figure S3 — The cumulative GC profile for prophage WOcauB2. (A) Gene presence in prophage WOcauB2. Colors of ORFs are as described in the legend of Figure 2. (B1) z′ curve for prophage WOcauB2. Segmentation points are marked with green squares. Segmentation point coincides with the HGT (B2gp46–B2gp47). (B2) The GC content distribution of prophage WOcauB2, using a 100 bp sliding window. [file Image3.PDF]

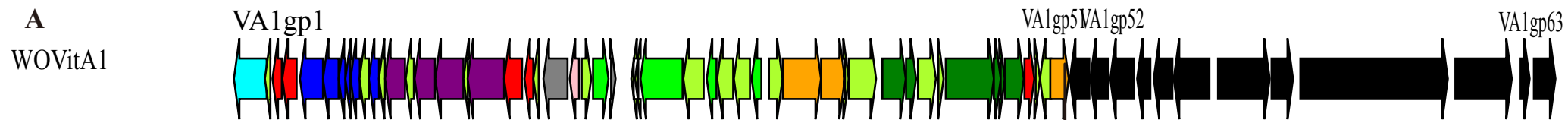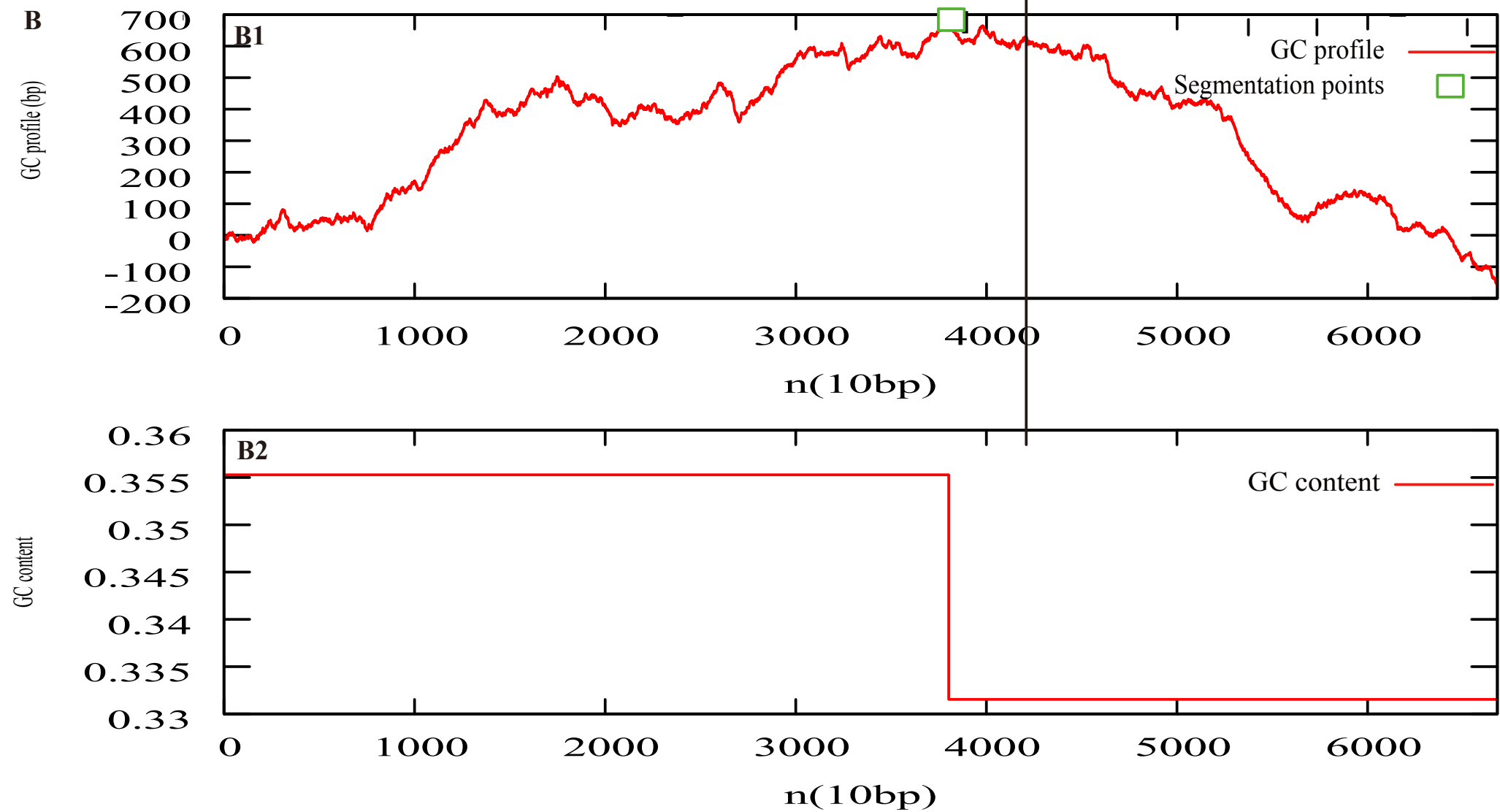

Supplement: Figure S4 — The cumulative GC profile for prophage WOVitA1. (A) Gene presence in prophage WOVitA1. Colors of ORFs are as described in the legend of Figure 2. (B1) z′ curve for prophage WOVitA1. Segmentation points are marked with green squares. Segmentation point includes the HGT (VA1gp52–VA1gp63). (B2) The GC content distribution of prophage WOVitA1, using a 100 bp sliding window. [file Image4.PDF]

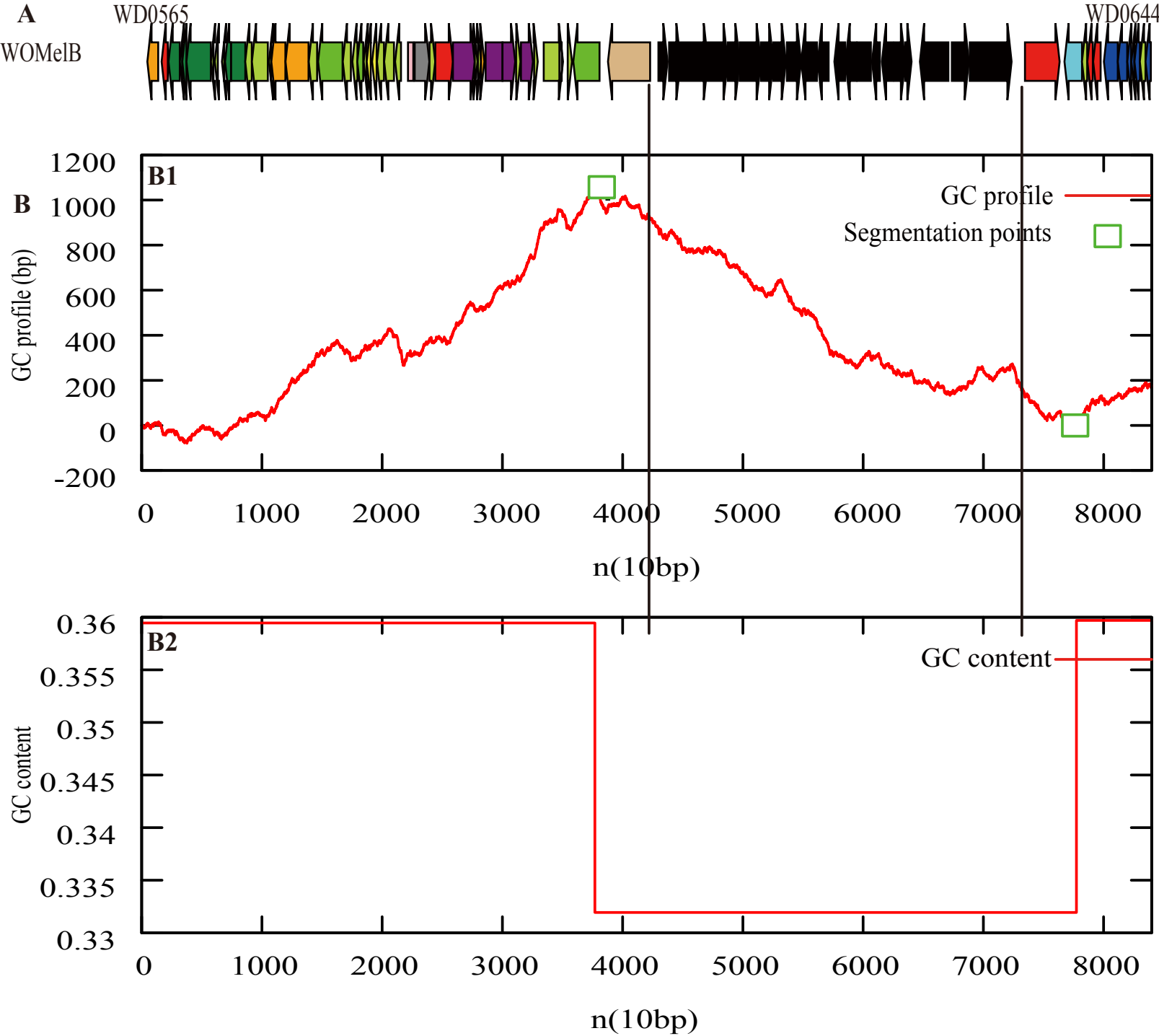

Supplement: Figure S5 — The cumulative GC profile for prophage WOMelB. (A) Gene presence in prophage WOMelB. Colors of ORFs are as described in the legend of Figure 2. (B1) z′ curve for prophage WOMelB. Segmentation points are marked with green squares. Segmentation point includes the HGT (WD0611–WD0632). (B2) The GC content distribution prophage WOMelB, using a 100 bp sliding window. [file Image5.PDF]
